# Accelerated breeding modernization: a global blueprint for driving genetic gains, climate resilience, and food security in rice

**DOI:** 10.1007/s00122-025-05060-1

**Published:** 2025-11-06

**Authors:** Sanjay K. Katiyar, Reshmi Rani Das, Lekha T. Pazhamala, Jérôme Bartholomé, Girish Chandel, Atugonza Bilaro, Maxwell Darko Asante, Khandakar Md Iftekharuddaula, Mirza M Islam, Ram Baran Yadaw, Ramlakhan Verma, Thati Srinivas, Chandra Mohan Yeshala, Herminio Abade, Viviane Raharinivo, Ruth Musila

**Affiliations:** 1 Genetic Diversity and Improvement, CGIAR-Africa Rice Center (AfricaRice), Bouake, 01 BP 2551 Côte d’Ivoire; 2 Rice Breeding Innovations, CGIAR-International Rice Research Institute (IRRI), Manila, Philippines; 3 Accelerated Breeding Initiative, Genetic Innovation, CGIAR-CIMMYT, Hyderabad, India; 4 CGIAR- International Center for Tropical Agriculture (CIAT), Cali, Colombia; 5 Indira Gandhi Krishi Vishwavidyalaya (IGKV), Raipur, India; 6 Tanzania Agricultural Research Institute (TARI), Kibaoni, Tanzania; 7 CSIR-Crops Research Institute (CRI), Kumasi, Ghana; 8 Bangladesh Rice Research Institute (BRRI), Gazipur, Bangladesh; 9 Bangladesh Institute of Nuclear Agriculture (BINA), Mymensingh, Bangladesh; 10 NARC- Nepal Rice Research Program (NRRP), Hardinath, Nepal; 11 ICAR- National Rice Research Institute (NRRI), Cuttack, India; 12 Acharya NG Ranga Agricultural University (ANGRAU), Maruteru, India; 13 PJ Telangana State Agricultural University (PJTSAU), Hyderabad, India; 14 Agricultural Research Institute of Mozambique (IIAM), Zambezia, Mozambique; 15 The National Center of Applied Research for Rural Development (FOFIFA), Antananarivo, Madagascar; 16 Kenya Agricultural and Livestock Research Organization (KALRO), Nairobi, Kenya; 17 Joint Research Unit for Genetic Improvement and Adaptation of Mediterranean and Tropical Plants (AGAP), CIRAD, UMR AGAP, Cali, Colombia

## Abstract

**Key message:**

ABM-BOx is a mission-critical transformation engine, built to fast-track genetic gains, boost climate resilience, and modernize outdated breeding programs into agile, data-driven, demand-responsive innovation platforms setting a global benchmark.

**Abstract:**

Rice plays a central role in global food security as climate threats continue to rise. Fast-tracking genetic gains and developing climate-resilient, market-preferred varieties require a bold, system-wide transformation of rice breeding practices worldwide. Baseline diagnostics of more than 25 national rice breeding programs across the Global South revealed critical bottlenecks: obsolete breeding strategy and scheme, fragmented workflows, limited technology access, and poor integration of seed system. This highlights the urgent need of breeding modernization to tackle rising food security risks. We introduce Accelerated Breeding Modernization-Breeding and Operational Excellence (*ABM-BOx*), a globally scalable framework to transform rice breeding programs into modern, data-driven, impact-oriented systems. *ABM-BOx* operationalizes a paradigm shift by translating the breeder’s equation into real-world impact through two synergistic engines: Breeding Excellence (BE) and Operational Excellence (OE). BE focuses on enhancing genetic gains through demand-driven breeding, strategic parental selection, recurrent population breeding, simulation-driven breeding scheme optimization, genomic selection, and predictive breeding. These strategies increase selection intensity, selection accuracy and shorten the breeding cycle. OE ensures speed, efficiency, and scalability through speed breeding-field based platforms, smart breeding-digital tools, breeding informatics-AI-powered decision tools, strategic costing-optimizing investments, and resilient seed systems. Additionally, Capacity Reinforcement and Functional Transformation-Accelerated Breeding Modernization (CRaFT-ABM) strengthens institutional capacity by focusing on talent, infrastructure, governance, and networks. More than a framework, *ABM-BOx* is a mission-critical transformation engine that drives innovation, speed, and impact to empower rice breeding efforts globally.

## Introduction

Rice feeds more than 3.5 billion people worldwide and accounts for more than 50% of the daily caloric intake in many Asian countries (Seck et al. 2012; Fang et al. 2021). Global rice consumption is expected to surge from 550 million tons (milled rice) in 2025 to nearly 584 million tons by 2050; driven by demographic expansion and economic growth across the Global South (Samal et al. 2022; FAO, 2025). In Africa, rice consumption is rising rapidly and is projected to more than double over the next 25 years, driven by urbanization, demographic momentum, and shifting dietary preferences (USDA FAS 2024). With consumption rising by 6% annually, rice is rapidly becoming a strategic staple in Sub-Saharan Africa’s food systems (FAO 2022). Cultivated on 11% of the world’s cropland, rice remains a cornerstone of global food and nutritional security (Kruseman et al. 2020). However, this vital role is increasingly at risk. The compounding pressures of climate change, population growth, and evolving dietary demands fueled by a global shift toward healthier, more diverse foods are exposing deep-rooted structural vulnerabilities in rice-based agri-food systems (FAO, IFAD, UNICEF, WFP, and WHO 2024).

Meeting the increasing demand and ensuring food security will require rapid deployment of high-yielding, climate-resilient, and widely adapted rice varieties. However, productivity in major rice-growing regions has stagnated (Rahman and Zhang 2023), constrained by outdated breeding pipelines, slow varietal turnover, and limited deployment of high-performing, resilient, and market-preferred varieties. Critically, genetic gains are not keeping pace with rising demand, widening the gap between current progress and future food security needs (Ray et al. 2013; Voss-Fels et al. 2019; Kusmec et al. 2021; Seck et al. 2023). Bridging this gap requires a rapid and radical transformation of rice breeding. Accelerated genetic gain, faster varietal turnover, and climate-smart innovations are imperative to strengthen food systems and meet the interconnected goals of SDGs 1 (No Poverty), 2 (Zero Hunger), and 13 (Climate Action) (United Nations 2023).

This article critically examines the emerging and systemic challenges of rice breeding programs across the Global South. In response, we present a transformative framework, *Accelerated Breeding Modernization-Breeding and Operational Excellence* (*ABM-BOx*) which re-engineers the breeder’s equation to fast-track genetic gains and enable the rapid, precise, and cost-effective delivery of market-preferred, climate-resilient rice varieties. For the first time, the rice breeding transformation agenda is strategically unified, comprehensively mapped, and fully operationalized, establishing a global benchmark for modernization. By integrating global science, breakthrough technologies, and streamlined breeding processes, *ABM-BOx* prioritizes speed, precision, and efficiency at the core of next-generation rice breeding.

## Strategic partnerships for advancing rice breeding and food security

Achieving sustainable food security in the Global South lies on the collaborative efforts of the National Agricultural Research and Extension Systems (NARES) and the Consultative Group on International Agricultural Research (CGIAR) research partnership. The CGIAR, with its extensive network and expertise, provides global science, resources, and technological innovations essential for accelerating genetic gains, while NARES serves as the critical force for local adaptation, evaluation, and varietal deployment. The following sections highlight the unique strengths of these key stakeholders and their sustained contributions to strengthening food security.

### NARES breeding: a cornerstone for global food security and SDG impact

For over six decades, CGIAR’s global rice breeding model has been deeply rooted in strong partnerships with public-sector NARES. These national breeding programs have been fundamental to agricultural transformation, driving rice productivity, enhancing production, and lifting millions out of poverty. In rice, many globally impactful mega-varieties have emerged from national programs, underscoring the vital role of local expertise, infrastructure, and adaptive capacity. In India, NARES-bred rice mega-varieties such as Swarna (MTU 7029), MTU 1010, MTU 1001, and BPT 5204 (Samba Mahsuri) collectively cover an estimated 8–10 million hectares across key rice-growing regions (Hossain et al. 2025; Merugumala et al. 2019). Similarly, in many countries across Asia and Africa, NARES-developed varieties dominate significant cultivation areas, underscoring their vital role in enhancing food security (Sonnino 2009; Adjah et al. 2022). NARES play a critical and irreplaceable role in generating localized market intelligence, identifying key market drivers, consumer preferences, and steering targeted multi-environment testing across diverse and remote target population of environments (TPEs). Their strategic role is vital for national varietal release and enabling effective delivery through strengthened adoption pathways. Anchored in conventional breeding strategies and practices, NARES rice breeding programs, for decades, remained closely aligned with global breeding efforts and played a pivotal role in enhancing food security and building breeding capacity across the Global South.

### CGIAR’s mission at a crossroads: tackling the global strategic challenge of food security

CGIAR is the world’s largest agricultural research partnership, driving innovative solutions to transform food, land, and water systems for a more sustainable and resilient future in the face of the climate crisis. Over the past decades, CGIAR-NARES partnerships have delivered significant welfare gains through elite genetics and improved breeding lines. Nearly 40% of modern varieties across all crops in the developing world emerged from these collaborations, generating an estimated USD 40 billion in annual economic surplus. Between 2022 and 2024, over 900 crop varieties were released in 66 countries, poised to reach 50–100 million people as scaling accelerates (CGIAR Initiative on Accelerated Breeding 2025).

In response to escalating global challenges, CGIAR’s Global Rice Program is leading a mission-critical transformation in rice research and innovation. This is being done through flagship efforts such as Breeding for Tomorrow (B4T) and the Accelerated Breeding Initiative (ABI) (CGIAR 2024a; CGIAR Initiative on Accelerated Breeding 2025). Breeding innovation is essential to boost genetic gains, close yield gaps, reduce input dependency, and rapidly deliver market-driven varieties to feed a projected 10 billion people by 2050 (FAO 2017). Over the past 5–7 years, CGIAR global rice breeding programs have been strategically overhauled and optimized, achieving greater impact per year and per dollar invested, while enhancing climate resilience and food security (Atlin and Econopouly 2022; Bhosale et al. 2025). CGIAR’s global rice breeding impact depends on empowered partnerships, particularly with NARES, as it has no mandate to release or disseminate varieties (CGIAR Initiative on Accelerated Breeding 2025). Improved genetics from CGIAR alone cannot drive transformation, the national context is critical. For real impact, science must align with country-specific priorities, strategies, and farmer needs. Lasting impact requires strategic collaboration, where empowered NARES partners lead in science, operations, and scaling. This integrated, systems-based approach connects breeding innovations with local solutions to drive real-world transformation.

**Fig. 1:**
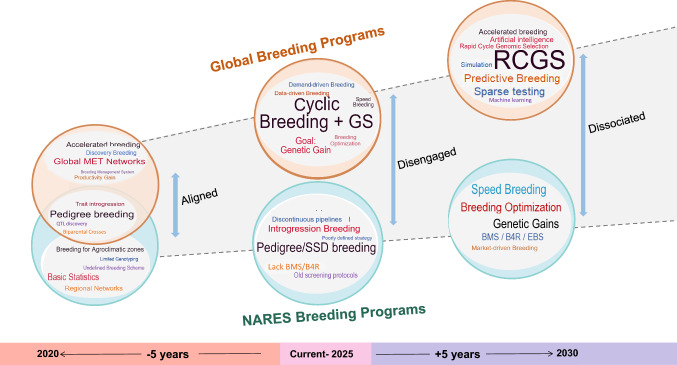
Evolving Dynamics Between CGIAR and NARES Rice Breeding Programs (2020–2030). The figure depicts the shifting alignment between CGIAR global rice breeding and the national programs visualized as word clouds over a decade. In 2020, both systems were closely aligned through shared methodologies and MET-driven evaluation. By 2025, CGIAR’s transition to cyclic, data-driven breeding powered by GS, digital tools, and BE-OE principles created a growing disconnect, as most NARES programs lag in modernization. Without urgent reforms, this gap may widen into structural dissociation by 2030. The figure highlights this trajectory and underscores the critical need to transform NARES programs through adoption of accelerated breeding modernization. Abbreviations: GS, Genomic Selection; MET, Multi-Environment Trial; BMS, Breeding Management System; B4R, Breeding 4 Results; EBS, Enterprise Breeding System; SSD, Single Seed Descent; QTL, Quantitative Trait Locus; NARES, National Agricultural Research and Extension Systems; CGIAR, Consultative Group for International Agricultural Research; BE, Breeding Excellence; and OE, Operational Excellence

The global mission to improve rice and ensure food security cannot advance without the parallel transformation of a vital partner, the public-sector NARES. Despite their critical role, the majority of national rice breeding programs in the Global South remain technologically outdated, fragmented, and slow to adopt modern breeding principles and enabling technologies. Falling behind the pace of global innovation and technological advancements, several NARES breeding programs are becoming increasingly disconnected from CGIAR-led efforts (Fig. 1). To close this gap, NARES must undergo accelerated breeding modernization to boost genetic gain, align with global initiatives, and deliver meaningful impact.

## NARES breeding systems: current realities and strategic pathways

**Fig. 2:**
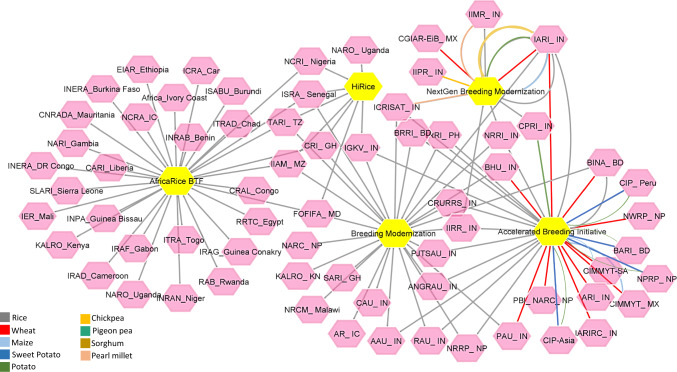
Catalyzing NARES Breeding Modernization in the Global South. The figure illustrates key strategic networks driving NARES breeding modernization through global initiatives; IRRI’s Breeding Modernization, CGIAR-EiB-NextGen Breeding, CtEH-HiRICE, and CGIAR-Accelerated Breeding Initiative. Yellow nodes indicate modernization platforms, pink nodes denote NARES partners, and edge colors reflect crop-specific collaborations. The network showcases strategic partnerships led by our team to accelerate breeding modernization across the Global South

NARES rice breeding programs are at a decisive crossroads. Outdated practices, fragmented systems, and chronic underinvestment are severely impeding their ability to deliver climate-resilient, high-performing varieties at the scale needed to address the challenges of an increasingly unpredictable climate change. Without significant reforms, these programs may hinder rather than facilitate agricultural transformation. Urgently needed are high-impact solutions to modernize NARES breeding programs, embedding global science and innovations, and strengthening their integration with the CGIAR–NARES breeding networks. This transformation is essential to reposition national breeding programs as agile, data-driven, and impact-focused platforms capable of delivering enhanced genetic gain. Recognizing the urgent need to transform NARES breeding programs, a strategic global network and platform for knowledge exchange, capacity building, and real-time support to implement “NARES Breeding Modernization – Breeding and Operational Excellence” has been established through CGIAR projects across South Asia and Sub-Saharan Africa (Katiyar et al. 2025; Fig. 2).

NARES programs are shaped by diverse agroecologies, geographies, mandates, capacities, and resources. It is, therefore, crucial to conduct context-specific assessments to identify their unique strengths, challenges, and opportunities for targeted transformation. Over the past 3–5 years, more than 25 NARES rice breeding programs across South Asia (India, Nepal, and Bangladesh) and Sub-Saharan Africa (Ghana, Senegal, Tanzania, Kenya, Mozambique, Madagascar, and Uganda) have been systematically assessed using the Breeding Program Assessment Tool (BPAT) and CGIAR’s Baseline Assessment Metrics. These expert-led diagnostics delivered actionable recommendations to enhance efficiency, accountability, and impact at both the national and regional levels. They provided deep insights into breeding strategies, germplasm pipelines, trialing systems, market orientation, mechanization, digitization, automation, technology access, data management, human resources, infrastructure, financial resources, and institutional performance (CGIAR Initiative on Accelerated Breeding 2025). The assessments identified each partner’s strengths, capacity gaps, and transformation goals. By benchmarking against global best practices, they uncovered critical system gaps and defined bold targets, shaping customized 5–10-year improvement plans to drive and sustain genetic gains. Continuous engagement with breeding teams has exposed persistent, systemic challenges, underscoring the critical need for unified, end-to-end modernization to drive scalable impact in rice breeding. (Text box 1).

## Text Box 1: Critical challenges undermining the NARES breeding systems

1. ***Lack of strategic focus***: The absence of demand-driven product profiles and forward-looking breeding strategies delays decision-making and hampers varietal replacement.
2. ***Obsolete breeding materials and methods***: Continued reliance on outdated crossing blocks and conventional parental selection constrains genetic diversity and innovation.
3. ***Inefficient breeding strategy and pipeline architecture***: Legacy pedigree breeding, poorly designed workflows, and non-optimized schemes extend breeding cycles and reduce output efficiency.
4. ***Weak trialing and evaluation systems***: Gaps in multi-environment testing, inconsistent data, and limited use of decision tools erode selection accuracy and confidence.
5. ***Limited use of modern breeding technologies***: Low adoption of speed breeding, smart breeding, digital technologies, precision phenotyping, and genomic selection slows progress and weakens genetic gains.
6. ***Disconnected and underpowered data systems***: Fragmented, paper-based systems with poor digitization, weak analytics, and limited decision support system use undermine real-time selection and forecasting.
7. ***Severe resource and capacity constraints***: Chronic infrastructure gaps, limited operational funding, and a shortage of expertise in quantitative genetics and digital breeding stall the modernization of breeding programs.
8. ***Poor integration with seed systems***: Weak feedback loops from farmers and limited coordination with seed value chains delay adoption and reduce the reach and relevance of improved varieties.

## Accelerated breeding modernization-Breeding and Operational excellence (*ABM-BOx*)

Achieving Zero Hunger under escalating food insecurity and climate pressures calls for a radical transformation of global and national rice breeding systems. Most NARES lack the integrated capacity to deliver scalable, high-performing varieties, and no simple technology fix can solve these deep-rooted challenges. However, cutting-edge innovations such as speed and smart breeding, genomic selection and predictive breeding, high-throughput phenotyping, breeding informatics, and AI-powered tools and decision support systems offer unprecedented potential to accelerate impact and deliver lasting change.

Driving a profound impact requires fast-tracking the adoption of breakthrough technologies and establishing data-driven, optimized rice breeding pipelines. NARES breeding programs must be repositioned to operate with greater speed, scale, and precision. What is needed is a bold, system-wide overhaul of the six decade old rice breeding paradigm, decisively moving away from legacy models toward a modernized, performance-driven approach tailored to empower national breeding programs. This requires a shift in institutional mindset and a fully integrated, enforceable unified framework that combines breeding excellence: modern principles, precision technologies, and data-driven selection with operational excellence anchored in globally validated best practices. Embedding this system-level transformation within national programs is critical to accelerate genetic gains, fast-track varietal replacement, and deliver climate-resilient, market-driven varieties. Such transformation requires reengineering breeding pipelines as optimized, data-led population improvement cycles designed to maximize genetic gain per year and per dollar, anchored in the core principles of the breeder’s equation (Seck et al. 2023).

The breeder’s equation, *R*_*t*_ = (*h*^*2*^ × *i* × *σp*)/*t*, quantifies the rate of genetic gain per unit time *R*_*t*_, where *h*^*2*^ represents narrow-sense heritability (selection accuracy), *i* is the selection intensity, *σp* is the genetic variance, and *t* is the breeding cycle length. Though simple in form, its execution demands strategic precision. Maximizing genetic gain requires deliberate action on three critical components, (i) improving *h*^*2*^ through superior trial design, replication, and data quality, (ii) increasing *i* by expanding selection intensity across broader, more competitive populations, and (iii) shortening *t* through speed breeding, genomic selection, and seamless transitions from evaluation to crossing. Genetic gain is not incidental; it needs to be systematically engineered.

**Fig. 3:**
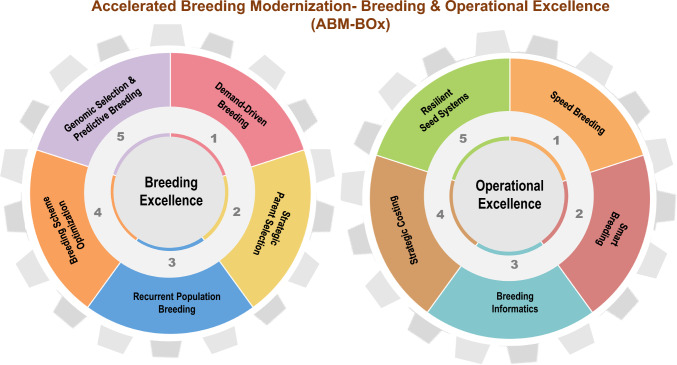
***ABM-BOx*: A Global Blueprint for Rice Breeding Transformation.** This figure illustrates *ABM-BOx* (Accelerated Breeding Modernization-Breeding and Operational Excellence), a transformative framework to modernize rice breeding into a high-performing, climate-smart engine for food security. It integrates two core pillars: Breeding Excellence, encompassing demand-driven breeding, strategic parent selection, recurrent population breeding, optimized breeding schemes, and genomic selection and Operational Excellence, featuring speed and smart breeding, breeding informatics, strategic costing, and resilient seed systems. Together, these domains offer a unified roadmap to modernize, scale, and future-proof rice breeding systems

We introduce *Accelerated Breeding Modernization-Breeding and Operational Excellence (ABM-BOx)*, an integrated, end-to-end framework to transform rice breeding globally. *ABM-BOx* is a unified platform for system-wide transformation, purpose-built to “Unbox Excellence” by embedding scientific rigor, modern breeding principles, breakthrough innovations, and world-class operational systems to deliver unmatched precision, speed, and efficiency across the breeding continuum. *ABM-BOx* systematically optimizes every element of the breeder’s equation through Breeding Excellence (BE) leveraging frontier science and advanced strategies and Operational Excellence (OE) integrating enabling technologies, tools, and global best practices (see Fig. 3). Central to this transformation is the synergy between BE and OE, which together engineer genetic gain with precision. BE enhances selection intensity, genetic variance, and accuracy via elite crosses, genomic selection (GS), recurrent population improvement, and optimized breeding schemes. OE complements this by shortening cycle time and boosting selection accuracy and efficiency through speed and smart breeding, digital tools, breeding informatics, and decision support systems. Together, they institutionalize precision across the pipeline and enhance gains compared to conventional systems. By aligning design, execution, and delivery, this BE-OE engine translates innovation into measurable impact. *ABM-BOx* accelerates sustainable genetic gain and ensures the rapid delivery of climate-resilient, market-aligned varieties at the scale and urgency demanded by the 21 st century food systems (Text Box 2).

## Text Box 2: Key drivers of accelerated breeding modernization

1. ***End-to-end optimization of breeding pipelines to accelerate genetic gain:*** Accelerated Breeding Modernization targets all elements of the breeder’s equation as an integrated system, addressing systemic inefficiencies rather than piecemeal fixes, to maximize the speed and impact of genetic improvement.
2. ***Demand-driven, TPP-anchored breeding:*** Breeding is anchored in stakeholder-validated target product profiles that ensure varietal development is demand-driven, climate-smart, and aligned for rapid replacement of obsolete varieties.
3. ***Data-driven decision-making:*** Replaces fragmented approaches with structured, performance-driven breeding pipelines powered by decision tools and simulation models, while AI leverages genotypic, phenotypic, and environmental data to enable precise predictions and efficient breeding decisions.
4. ***Mainstreaming genomics, speed, and smart breeding:*** Harnesses breeding innovation, genomic selection and predictive breeding, high-throughput phenotyping, speed breeding, smart breeding, shorten cycles, optimize resources, and improve selection accuracy.
5. ***System-wide institutional and cultural reform:*** Drives a paradigm shift from fragmented, breeder-centric approaches to integrated, collaborative, and digitally enabled systems anchored in global best practices to achieve breeding and operational excellence.

### Accelerated breeding modernization-Breeding Excellence (BE)

Breeding Excellence (BE) acts as the scientific foundation of the *ABM-BOx* framework, which is specifically designed to accelerate genetic gain through the integration of modern breeding principles, scientific rigor, and data-driven decision-making. It includes demand-driven breeding, strategic parent selection, recurrent population breeding, breeding scheme optimization, genomic selection, and predictive breeding. The following sections outline the key pillars that define Breeding Excellence.

#### Demand-driven breeding: defining breeding targets with market insights

For decades, public NARES breeding programs have lacked access to market intelligence, value chain insights, and product design tools, resources that demand-driven private sector breeding routinely leveraged. Lacking real-time data on stakeholder needs, these programs prioritized scientific outputs over market relevance, thus limiting their impact.

Demand-driven breeding is a transformative shift in public breeding systems, repositioning market intelligence as the central driving force behind varietal development. By aligning breeding priorities with the explicit demands of stakeholders, including farmers, consumers, and value chain actors, it enhances both the technical merit and the likelihood of adoption of new varieties. Modeled on private sector efficiency, this approach redefines breeding pipelines as market-responsive enterprises, engineered for relevance, scalability, and measurable impact. Central to this model is the Target Product Profile (TPP), a trait-based blueprint that defines the ideal variety for specific, high-impact market segments. These market segments, shaped by agroecology, grain quality, culinary preferences, and socioeconomic drivers, guide precision breeding toward varieties with real-world value (Donovan et al. 2022; Custodio et al. 2023; Friedmann et al. 2024).

Modern breeding strategically aligns market segments, target product profiles, and breeding pipelines to maximize impact. Platforms such as The Global Market Intelligence Platform (GloMIP; https://glomip.cgiar.org/) enable the development of TPPs (CGIAR 2023) sharpening trait prioritization, ensuring product-market fit, and guiding resource allocation across pipelines. Demand-driven breeding improves adoption rates, strengthens stakeholder engagement, and enhances varietal differentiation. TPPs operationalize market demand into clear breeding objectives by prioritizing essential and value-added traits that accelerate varietal adoption. Developed through structured engagement with farmers, millers, traders, exporters, and consumers, TPPs ensure breeding outputs are agronomically robust and commercially viable. To maintain relevance, TPPs must be regularly updated to reflect evolving market preferences, production constraints, and consumer expectations. Each TPP serves as an implementation guide, directing its corresponding breeding pipeline toward the deliberate delivery of high-impact, demand-driven products. In the past 3 years, a critical shift has begun. Select NARES programs have begun adopting demand-driven breeding, product designing tools and technologies, aligning varietal development with demand, accelerating adoption, and driving greater impact (CGIAR Initiative on Accelerated Breeding 2025). Embedding demand-driven breeding within *ABM-BOx* anchors rice breeding in real-world relevance.

#### Strategic parental selection: optimizing crossing blocks for genetic gain

Identifying elite founder parents is the cornerstone of any effective breeding program (Allard 1999; Falconer 1996). Elite germplasm comprises genotypes enriched with favorable alleles that confer high breeding value, defined by the average performance of progeny in the target environments (Juma et al. 2021). Genetic gain for grain yield remains unacceptably low in many public NARES rice breeding programs across the Global South. This is driven by long breeding cycles and a continued dependence on traditional landraces, outdated varieties, and unimproved trait donors, often chosen as parents based solely on phenotype. Furthermore, oversized and non-strategic crossing blocks, sometimes involving 300–700 parents, are deployed across pipelines without alignment to defined product profiles. This lack of targeted parental selection has diluted breeding focus, reduced efficiency, increased costs, and constrained genetic progress.

Despite major advances in molecular breeding, parental selection in most national programs continues to rely primarily on phenotypic evaluation. Breeders often rely on visual scores and trait averages, such as yield, grain quality, and disease resistance, rather than leveraging genetic merit, genomic predictions, or genomic estimated breeding values (GEBVs). This intuition-based approach undermines the precision of crossing decisions and significantly constrains the acceleration of genetic gain. Elite-by-elite crossing emphasizes parental selection based on genetic merit using GEBVs (Juma et al. 2021). Derived from pedigree or genome-wide marker data, GEBVs capture the additive genetic component, the heritable fraction most critical for sustained genetic gain. Unlike best linear unbiased prediction (BLUP), which reflects total phenotypic performance including non-additive effects, GEBVs offer a more precise measure of true breeding potential. While BLUPs are useful for selecting top performers within environments, GEBVs enable forward-looking, data-driven crossing decisions (Chung and Liao 2020; Cobb et al. 2019). Integrating GEBVs into public NARES rice breeding is essential to identify truly elite parents and accelerate long-term genetic gains. Recent evidence also shows that optimizing the number of elite parents using complex trait indices significantly enhances the likelihood of higher genetic gain (Covarrubias-Pazaran et al. 2022).

NARES programs need to transition to a modern parental selection strategy, where parents are selected based on breeding values (BVs) derived from pedigree or genomic data. These values are estimated from multi-environment performance and genome-wide marker data using a genomic relationship matrix (GRM). The high-ranking genotypes thus identified should then be incorporated in pipeline-specific crossing blocks that are aligned with defined Target Product Profiles (TPPs). Since these elites are already genotyped, they can be further analyzed to assess favorable alleles, trait diversity, and any existing genetic gaps. This genomics-enabled, strategically designed crossing block forms the foundation for elite-by-elite quantitative genetics-based recurrent selection. This approach is crucial for driving genetic gain with greater precision and efficiency.

#### Recurrent population breeding: unlocking sustained genetic gain

Historically, NARES rice breeding programs have relied on pedigree-based methods that significantly contributed to the past yield gains. However, these approaches have been inherently slow, labor-intensive, cost-inefficient, and not optimized for maximizing the genetic gain per unit time, a critical metric for long-term breeding success. In contrast, recurrent selection represents a paradigm shift, particularly for complex polygenic traits such as yield. Recurrent selection offers a strategic framework that decouples population improvement from product development, enabling parallel optimization and faster genetic gain through accelerated turnover and targeted line or hybrid development (Gaynor et al. 2017; Cobb et al. 2019; Seck et al. 2023). This structural clarity is central to modernizing breeding pipelines and maximizing the return on investment. For NARES, deploying tailored recurrent selection schemes integrated with innovations such as modified single seed descent (SSD), speed breeding, high-throughput genotyping and phenotyping, and genomic selection, is essential. These tools collectively shorten breeding cycles, enhance selection precision, and improve adaptability to climate change and evolving market demands. Even with limited resources, programs can achieve significant gains by rapidly cycling and selecting parents with high-breeding value. The following sections outline the three core features that consistently define successful breeding programs.

##### Population improvement—a catalyst for accelerated genetic gain

Population improvement is the cyclical advancement of a breeding population derived from elite founder lines, driven by the systematic selection of parents based on the breeding value or genotypic merit for complex quantitative traits. In each cycle, segregating populations are developed, superior recombinants are selected, and inter-mated to generate the next generation. This iterative process shifts allele frequencies toward favorable variants, progressively enhancing the population mean of derived lines, hybrids, or clones (Atlin et al. 2017a; 2017b; Rutkoski 2019a; 2019b). This approach has been widely adopted in high-performing private sector programs, to deliver higher and more sustained genetic gains (Dai et al. 2015; Boyer et al. 2013). While many breeding programs continue to rely on phenotypic selection or raw genotypic scores to select founder lines, compelling evidence indicates that breeding values derived from pedigree or genomic data consistently delivered superior long-term genetic gain (Piepho et al. 2008). This continuous loop is the core engine of adaptive breeding, crucial for building resilience under increasing climate variability.

To accelerate genetic gain and drive the directional improvement of NARES breeding programs, early and strategic recycling of elite lines is essential. This strategy reduces generation intervals, increases selection intensity, and accelerates allele turnover, collectively enhancing breeding speed, efficiency, and adaptability. Two key interventions can enable this transformation: (i) integrating single seed descent (SSD) with speed breeding to rapidly fix lines and (ii) implementing Rapid-Cycle Genomic Selection from Stage 1 (the first multi-environment testing) performance trials, to identify superior individuals for immediate reuse as parents in the next cycle.

##### Product development—accelerated delivery of improved varieties

Alongside population improvement, commercial cultivar selection aims to identify advanced lines or families, often shared with recombination sets, that exhibit strong potential for varietal release. These candidates undergo rigorous multi-environment trials (METs) across their target population of environments (TPEs), with selection based on trait stability, broad adaptation, and market relevance. This process ensures the delivery of climate-resilient, high-performing varieties that are aligned with the demands of farmers and consumers.

##### Rapid-cycle breeding—accelerating climate resilience

Plant breeding is the most effective strategy for climate adaptation in agriculture. While gene with large effect for drought, submergence, heat, and cold tolerance is valuable, the traits most critical for long-term resilience, such as phenology, yield stability, and stress tolerance, are polygenic. Improving these complex traits requires continuous shifts in allele frequency across many loci, which is achievable only through recurrent population breeding that delivers a steady stream of superior recombinants and cultivars (Atlin et al. 2017a; Li et al. 2018a).

A climate-ready breeding pipeline must integrate three essential elements: access to elite germplasm across geographies, shortened breeding cycles, and rigorous multi-environment testing that reflects real-world climate variability. Cultivars must be evaluated across the full range of stress conditions they will face during their commercial lifespan to ensure performance, resilience, and stability (Atlin et al. 2017a). Adaptation depends not only on population design, but also on speed. The ability to rapidly develop, test, release, and replace varieties determines how effectively improved genetics translate into impact. However, breeding cycles in most NARES rice programs across the Global South still exceed 10 years, constraining genetic gain, delaying varietal turnover, and weakening climate resilience. Halving the cycle time can double the rate of genetic gain and increase recombination, improving the chances of capturing favorable haplotypes suited for dynamic environments. This agility is critical not just for climate adaptation, but to stay ahead of rapidly evolving pests and pathogens. In the era of climate disruption, rapid-cycle breeding for NARES is not optional, it is the backbone of future-proof agriculture.

#### Breeding scheme optimization: engineering genetic gains by design

Optimized breeding schemes are the foundation of accelerated genetic gain, faster product delivery, and improved cost-efficiency. Unlocking their full potential requires transforming breeding pipelines into structured, process-driven systems through continuous optimization of crossing, evaluation, and selection (Covarrubias-Pazaran et al. 2021, 2022). This shift demands more than incremental improvements, it requires rigorously designed, near-optimal strategies that clearly surpass current practices by enhancing gain, product quality, or reducing costs. Structured breeding schemes offer a rigorous, data-driven framework to design, execute, and optimize breeding programs, enabling breeders to manage complexity, assess progress, and make informed, strategic choices. However, most NARES rice breeding programs currently lack such a disciplined approach, instead relying on fragmented, ad hoc decisions that limit efficiency, consistency, and long-term impact. In resource-constrained public breeding systems, breeding scheme optimization is a game-changer for maximizing the genetic gain per dollar. Instead of demanding large investments, it leverages simulation-driven insights to fine-tune key variables, such as parental selection, progeny size, trial design, and selection intensity across the pipeline (Gaynor et al. 2017; Bassi et al. 2016).

Optimizing breeding schemes requires robust tools to balance key parameters such as number of parents, crosses, progeny sizes, resource allocation, and selection intensity, across the breeding cycle. These interdependent decisions shape genetic gain and must be fine-tuned through a structured, data-driven approach (Henryon et al. 2014; Yabe et al. 2017; Cobb et al. 2019; Pook et al. 2025). A well-planned scheme defines the crossing design, evaluation strategy, and selection decisions, specifying whom to cross, how to test, and what to select (Covarrubias-Pazaran et al. 2022). Table 1 captures an optimized breeding framework structured around three core domains, namely, crossing design, evaluation strategy, and selection decisions, highlighting critical drivers for accelerating genetic gain. These components collectively drive efficiency, accelerate gain, and ensure program impact. For resource-limited public breeding programs, the cost per unit of genetic gain is now a key performance metric, guiding smarter investments. Simulation tools further empower breeders (Kusmec et al. 2021; Seck et al. 2023) to test alternatives virtually, reducing risk and enabling data-backed, cost-effective decisions. Breeding simulations powered by AlphaSimR, a state-of-the-art tool, have enabled rice breeding programs to optimize breeding schemes with greater precision, efficiency, and predictive power (Gaynor et al. 2021). In brief, optimized breeding schemes are the engine of modern breeding programs, streamlining decisions and resources to consistently deliver greater genetic gains with precision and efficiency.

**Table 1:** Breeding scheme optimization: strategic decision domains to maximize genetic gain

Decision domain	Components	Key considerations	Driver of genetic gain
**1. Crossing design**	Selection and number of parents	• Genetic diversity • Elite by genetics • Breeding objective (TPP)	• Enhances selection intensity • Maximizes genetic gain
	Crossing combinations	• Trait complementarity • Combining ability • TPE alignment	• Favorable allele stacking • Enhances selection accuracy
	Progenies per cross	• Capture rare recombinants • Multi-trait selection • Genomic selection support	• Enhances selection intensity • Improves selection accuracy
	Mating design	• Structured genetic variation • Maximum impact • Genomic selection support	• Increases selection efficiency • Enhances selection intensity
**2. Evaluation strategy**	Trail locations	• Target population of environments (TPEs) representation • G X E interaction • Heritability of traits	• Enhances selection intensity • Improves selection accuracy
	Replications	• Trait variability and heritability • Trial design • Genotype number and plot size	• Improves data reliability • Improves selection accuracy
	Check entries	• Benchmark for comparison • Stability assessment • Across-site comparisons	• Enhances selection accuracy • Enables reliable G × E analysis
	Experimental design	• Reduce experimental error • Ensure statistical validity • Facilitate multi-location comparisons	• Improves selection accuracy • Better G × E analysis
	Plot configuration	• Trait expression • Minimize border effects • Improve field operation and precision	• Enhances selection intensity • Improves selection accuracy
**3. Selection Decisions**	Selection method	• Selection indices and Breeding goals • Enable early selection • High accuracy	• Drives selection accuracy • Maximizes genetic gain
	Selection unit	• Specify target: Individuals/families/rows • Breeding stage and Pipeline structure • Trait heritability	• Drives selection accuracy • Maximizes genetic gain
	Selection intensity	• Breeding stage and pipeline structure • Trait heritability • Selection method	• Enhances selection intensity • Maximizes genetic gain
	Breeding cycle time	• Stage-specific selection strategy • Speed breeding technologies • Speed vs. accuracy	• Enhances selection intensity • Maximizes genetic gain

#### Genomic selection and predictive breeding—driving breeding efficiency forward

Genomic selection (GS) is redefining crop breeding by delivering faster and more accurate genetic gains than conventional approaches. By combining genotypic data, high-quality phenotyping (HTP), trait-linked markers, and predictive algorithms, GS enables the precise estimation of GEBVs, even for complex traits, allowing early selection of superior lines as both products and parents (Meuwissen et al. 2001; Seck et al. 2024). In rice, GS has gained significant traction in the recent past, driving more targeted selection and tighter alignment with trait-specific breeding goals (Bartholomé et al. 2022). Breeding programs are increasingly using GS to accelerate varietal advancement through: (1) earlier recycling of elite parents, (2) screening of larger populations to uncover optimal trait combinations, and (3) prioritization of top candidates for field testing. Together, these strategies enhance selection intensity, shorten breeding cycles, and improve genetic gain. Although simulations forecast significant gains (Gaynor et al. 2017; Gorjanc et al. 2018), real-world field validation remains limited. To fully unlock its potential, GS must be embedded within programs equipped with robust phenotyping, cost-effective genotyping, and seamless data integration (Voss-Fels et al. 2019). However, the realized impact of GS depends on trait architecture, genotyping costs, and institutional capacity.

The widespread adoption of GS in rice is now possible due to affordable, high-throughput genotyping platforms. SNP arrays and next-generation sequencing (NGS) technologies enable rapid and accurate genotyping of large populations. Platforms such as 1KRiCA (1 K Rice Custom Amplicon) provide targeted SNP profiles (~ 1000 markers, including over 90 trait-linked loci) for under $10/sample, making GS deployment scalable and cost-effective across breeding pipelines (Arbelaez et al. 2019). These tools generate rich genomic datasets that, when coupled with high-resolution phenotyping, enable informed, data-driven decisions at every stage of selection. As genotyping costs declined and computational analytics advanced, GS has emerged as a core engine of breeding efficiency, delivering faster genetic gains without increasing program size (Nguyen et al. 2023; Cobb et al. 2019; Bernardo 2020). Concurrently, the convergence of high-density genotyping, HTP, and advanced remote sensing is transforming field evaluation, enabling non-invasive, high-resolution trait capture at scale and ushering in a new era of predictive, data-driven breeding.

##### Enabling technologies and strategic applications of genomic selection (GS)

Genomic selection is rapidly transforming breeding, especially when integrated with a suite of enabling technologies that boost accuracy, efficiency, and scalability. These include, a) cost-effective genotyping platforms such as 1KRiCA (Arbelaez et al. 2019); b) high-throughput phenotyping (HTP) and remote sensing, which enable rapid, non-destructive trait measurement at scale, turning phenotyping into a powerful, cost-efficient engine for selection (Desta and Ortiz 2014; Lobos et al. 2017); and c) speed breeding innovations, including Field-RGA, field nurseries with recurrent population improvement, and SSD. When combined with GS, these approaches enable continuous allele recycling and directional genetic gain for complex, climate-resilient traits (Atlin et al. 2017a; Voss-Fels et al. 2019).

GS empowers several high-impact applications, such as i) early parental selection using genomic estimated breeding values (GEBVs) improves the probability of passing on favorable alleles (Mrode 2014); ii) sparse phenotyping, where a strategic subset of lines is tested across key environments, which amplifies selection intensity and captures G × E interactions through advanced GS models (Jarquin et al. 2020; Endelman et al. 2014); iii) rapid recycling of early-stage candidates based on GEBVs shortens breeding cycles, and iv) cross optimization through genomic prediction of progeny mean and within-family variance enables informed cross designs for both immediate and sustained genetic gain. Collectively, these advances are driving a fundamental shift toward predictive, accelerated, and data-driven breeding pipelines (Clark and van der Werf 2013; Lehermeier et al. 2017; Gorjanc et al. 2018; Kusmec et al. 2021).

### Accelerated breeding modernization—Operational Excellence (OE)

Operational Excellence (OE) ensures that scientific innovations in breeding are implemented with precision, efficiency, and real-world relevance. OE integrates globally recognized best practices, advanced technologies, digital tools, and support services into a unified delivery platform. The key pillars include speed breeding, smart breeding, breeding informatics, strategic costing, and resilient seed systems, each designed to equip NARES programs to evolve into high-performing, modern breeding systems. Operational Excellence (OE) plays a critical role in delivering climate-resilient, farmer-preferred rice varieties by elevating breeding operations into scalable, high-efficiency systems. The following sections provide a detailed overview of OE’s key components.

#### Speed breeding: the most powerful driver of genetic gain

Among all the variables in the breeder’s equation, shortening the breeding cycle is the most practical, cost-efficient, and high-impact strategy to accelerate genetic gain. Despite this, over 80% of public-sector NARES breeders continue to rely on slow and inefficient, conventional pedigree breeding, extending variety development timelines to 12–15 years and delaying the impact at scale (Lenaerts et al. 2018). Especially in low-resource settings, there is an urgent need to adopt scalable, low-cost, and high-return interventions. Speed breeding strategies such as off-season nurseries, rapid generation advancement (RGA), field-based RGA (F-RGA), field nursery (F-N), and double haploids (DH) have proven effective in compressing timelines in rice breeding (Collard et al. 2017, 2019; Lenaerts et al. 2018; Ahmed et al. 2022). While conventional approaches typically require 7–8 years to achieve homozygosity and line fixation, optimized RGA can accomplish this from F₂ to F₅/F₆ within just 2 years, substantially shortening breeding cycles by 3 years or more (Collard et al. 2017, 2019; Tanaka et al. 2016).

**Fig. 4:**
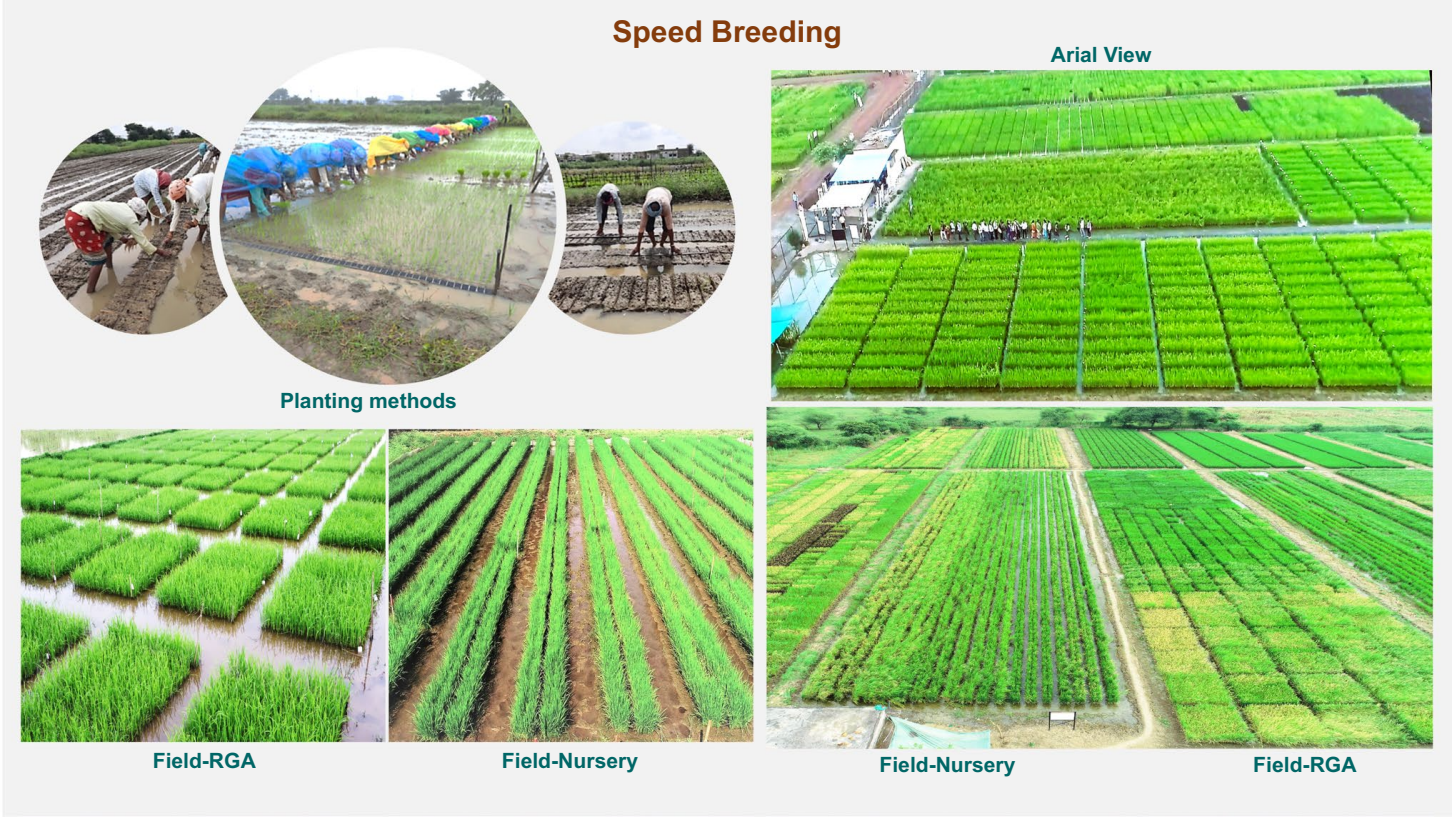
Speed Breeding Technologies to shorten breeding cycle. This figure highlights scalable, field-based speed breeding solutions tailored for NARES. It showcases diverse planting methods, optimized layouts, and widespread deployment of Field-RGA (F-RGA) and Field Nurseries (F-N). Aerial views reveal the scale, efficiency, and adaptability of these innovations in accelerating generation turnover and expanding breeding capacity

Achieving faster genetic gain requires an integrated rapid-cycle approach that includes advancing progeny through SSD, deploying scalable speed breeding tools such as RGA, Field-RGA, and Field-Nursery and selecting parents using GEBVs (Fig. 4). These innovations enable 3–4 generations per year, substantially reducing the time required to develop fixed lines to under 2 years and are cost-efficient (Table 2 and Table 3). Widely adopted across South Asia, these NARES-friendly, cost-effective, and infrastructure-light speed breeding innovations have transformed rice breeding pipelines. They reduce cycle time by up to two- to three-fold, improve resource-use efficiency, increase throughput exponentially, require minimal training, and no infrastructure investment (International Rice Research Institute 2022). For national programs with budget constraints and mounting demand, speed breeding provides a game-changing, resource-efficient strategy to fast-track genetic gains and enhance breeding impact.

**Table 2:** Speed breeding: strategies to accelerate impact

Impact area	Strategies
Breeding cycle compression	Accelerate generation turnover to 3–4 cycles per year Replace slow pedigree methods with SSD for rapid fixation Streamline early-stage testing to fast-track parent recycling
Boosting selection intensity and accuracy	Increase population size to maximize genetic variance Intensify selection pressure to capture elite performers Expand multi-environment testing with higher replications for greater accuracy

**Table 3:** Empowering national programs: key features of scalable speed breeding innovations in rice

Technology combination	Generations per year	Key features	Advantages
**SSD + RGA** (Controlled environment)	3.5–4.0	In glasshouse, with temperature, light and humidity control; nutrient stress	Multiple generations per year
**SSD + Field RGA **(F-RGA)	2–3.5	Ultra-dense spacing (5 × 5 cm) under natural field conditions; competitive conditions and nutrient stress	High-throughput, > 15–20X efficiency, no infrastructure required
**SSD + Field Nursery** (FN)	2–3.5	Field-grown panicles on raised beds; competitive conditions and nutrient stress	High-throughput, low mortality, efficient, no infrastructure required

#### Smart breeding: integrating digital tools and analytics for informed decisions

**Fig. 5:**
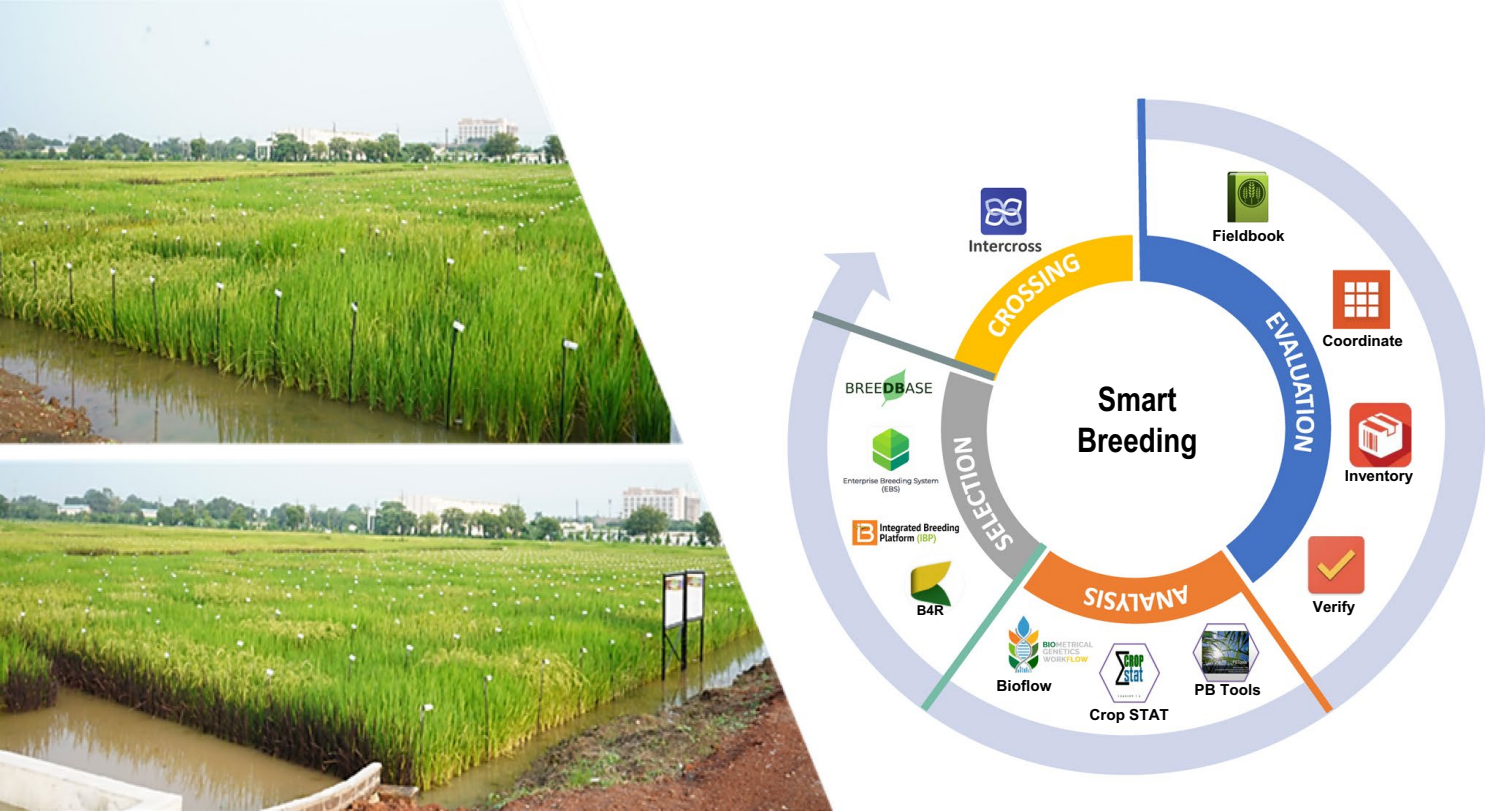
Smart Breeding: Digital Integration for Accelerated Genetic Gains. The figure highlights digital tools transforming rice breeding precision and efficiency. The left panel shows wide scale barcoding and digitization in NARES field trials. The right panel illustrates an end-to-end smart breeding workflow—from crossing to selection—leveraging PhenoApps, analytics platforms, and decision support systems to enable real-time data capture, rapid analysis, and informed selection, significantly boosting genetic gains

Smart breeding integrates cutting-edge digital innovations, including bar coding, automation, real-time phenotyping, integrated data management, and advanced analytics to revolutionize selection accuracy and breeding efficiency (Fig. 5). The broad adoption of (i) Decision support systems, (ii) PhenoApps, and (iii) Breeding analytics tools has transformed data capture, analysis, and interpretation, enabling faster, more informed decisions and significantly accelerating genetic gains (Rebetzke et al. 2018; Yang et al. 2020; Jangra et al. 2021).

##### Decision support systems

Breeding data management and decision support systems are critical to modern, data-driven rice breeding. They enable the seamless capture, integration, and analysis of complex breeding datasets, which include phenotypic, genotypic, environmental, and pedigree information. By transforming data into real-time, actionable insights, these systems facilitate strategic decision-making, enhance selection accuracy, optimize breeding cycles, and enable faster genetic gain across breeding programs. Commonly available breeding management systems are (i) The Enterprise Breeding System (EBS; https://excellenceinbreeding.org/toolbox/tools/enterprise-breeding-system-ebs), an innovative, open-source platform developed by CGIAR that integrates germplasm, nursery, and field trial management in a centralized, data-driven framework. EBS integrates advanced analytics and decision support tools to streamline workflows, enable real-time data access, and standardize breeding operations. By consolidating various tools into a single system, EBS empowers breeders to achieve genetic gains through faster, smarter, and more efficient breeding decisions, and (ii) The Breeding Management System (BMS; https://excellenceinbreeding.org/toolbox/tools/breeding-management-system-bms), developed by the Integrated Breeding Platform (IBP), is a web-based solution designed to enhance the efficiency, accuracy, and security of data across breeding programs. BMS offers comprehensive tools for data capture, analysis, and real-time tracking, facilitating seamless integration and streamlined decision-making throughout the breeding pipeline.

##### PhenoApps

PhenoApps integrates image analysis, affordable sensors, and machine learning to create next-generation mobile tools for breeding. By combining real-time data capture, processing, and analytics in a user-friendly interface, PhenoApps streamline phenotyping, enhance data quality, and accelerate genetic gain. The most widely used is Field Book, an intuitive PhenoApp that facilitates field data collection by enabling real-time entry, minimizing transcription errors, and speed up data analysis (Rife and Poland 2014). Its high customizability allows breeders to configure trait lists, scoring scales, and metadata to suit specific breeding objectives. Seamless integration with platforms such as the Breeding Management System (BMS) and Enterprise Breeding System (EBS) supports streamlined end-to-end breeding workflows. Complementary PhenoApps such as Intercross (cross management), Coordinate (customized data collection), Prospector (NIRS-assisted data collection), OneKK (seed weight and trait measurement), and Verify (barcode-enabled logistics) enhance precision, streamline field operations, and improve the overall efficiency of breeding programs.

##### Breeding analytics tools

Biometrical Genetics Workflow (BioFlow; https://cgiar-market-intelligence.shinyapps.io/bioflow/), developed by CGIAR, is an integrated breeding analytics and decision support tool designed to accelerate selection, shorten breeding cycles, and boost genetic gain. It supports spatial analysis, heritability estimation, best linear unbiased estimators (BLUEs), best linear unbiased predictors (BLUPs), and single trial analysis, while enabling multi-environment trial evaluation, selection index computation, genetic gain monitoring, and optimal cross-selection; empowering high-efficiency data-driven breeding decisions. BioFlow integrates multi-source data, enabling breeders to evaluate test genotypes, recycle elite parents, and identify candidates for advancement. Its modular design supports precise genetic evaluations, driving greater efficiency, accuracy, and impact in breeding programs. Plant Breeding Tools (PBTools; http://bbi.irri.org/), developed by IRRI, is a user-friendly statistical software package for the design, analysis, and interpretation of breeding trials. It supports large-scale datasets, offering tools for exploratory analysis, G × E interaction, variance partitioning via mixed models, yield evaluation, and genetic value prediction using BLUPs, all presented through an intuitive interface with clear tables and graphs (Henderson 1975; Gilmour et al. 1997; Piepho et al. 2008). Other analytics tools used in rice breeding include Crop-STAT for breeding trial evaluation and STAR for statistical analysis, all of which drive precision and efficiency across breeding pipelines.

Digitization platforms such as EBS, BioFlow, and PhenoApps (e.g., Field Book) have transformed fragmented breeding workflows into integrated, data-driven ecosystems. Barcode tracking, automated trials, and cloud-based storage enable real-time, high-quality phenotyping and data-driven selection (CGIAR Excellence in Breeding 2023; CGIAR Initiative on Accelerated Breeding 2023).

#### Breeding informatics: leveraging AI and ML in rice breeding

Artificial intelligence (AI), machine learning (ML), and deep learning (DL) are transforming crop breeding by accelerating data analysis, improving precision, and enabling faster, smarter decisions (MacNish et al. 2025; Crossa et al. 2025; Eftekhari et al. 2024; Yoosefzadeh-Najafabadi et al. 2023; Cheng and Wang 2024). In recent years, the integration of these tools, alongside bioinformatics and high-performance computing, has become widely adopted in plant breeding programs. These technologies allow breeders to leverage high-throughput phenotyping, predict phenotypes, breeding values and G × E interactions, as well as forecast yield (Table 4). Cutting-edge platforms also support trial design and streamline genomic selection pipelines (Xu et al. 2022; Bhat et al. 2023).

**Table 4:** Breeding informatics: leveraging AI and ML applications in crops

Application	Crop	Description	References
Genomic prediction	Rice	Deep learning (DeepCCR) achieved 63.3%–78.2% genomic prediction accuracy across eight traits	Ma et al. (2024)
	Rice Maize Wheat	TrG2P DL models outperformed seven top GS tools, enhancing yield prediction by 39.9% (rice), 6.8% (maize), and 1.8% (wheat)	Li et al. (2024)
	Wheat	DL models (MLP, CNN) predicted five quantitative traits with up to 5% higher accuracy than standard GS models	Sandhu et al. (2021)
	Rice	TOP ML algorithm reached 91% accuracy in identifying phenotypic matches to the target ideotype	Yang et al. (2022)
Yield prediction	Rice	ANN-based ML model predicted yield of mechanically transplanted rice with an RMSE of 4.57	Basir et al. (2021)
	Rice	Of six AI models tested, hybrid models best predicted rice production across China	Mokhtar et al. (2024)
	Rice	Bayesian ML algorithms using UAV remote sensing predicted rice yield with a correlation of 0.958	Sarkar et al. (2024)
	Rice	Hybrid learner “RaNN” achieved 98% correlation between actual and predicted yield using ML methods	Lingwal et al. (2022)
Phenotype prediction	Soybean	CNN-based DL accurately predicts phenotypes from SNPs, bypassing genotype imputation	Liu et al. (2019)
	Wheat	DeepGS, a DL neural network, complements RR-BLUP for predicting phenotypes from genotypes	Ma et al. (2018)
Breeding program optimization	Maize	AutoGP (http://autogp.hzau.edu.cn) streamlines breeding by enabling faster, more accurate genomic selection	Wu et al. (2025)

ANN, artificial neural network; CNN, convolutional neural network; MLP, multilayer perceptron; RaNN, Random Forest in-combination with the multilayer Feedforward Neural Network; RMSE, root-mean-square error; RR-BLUP, Ridge Regression-Best linear unbiased prediction BLUP TrG2P, A transfer learning-based tool; TOP, target-oriented prioritization; and UAV, unmanned aerial vehicle

##### ML- and DL-driven GS models enhance prediction

The steep drop in genotyping costs has enabled large-scale marker discovery, making genomic selection accessible even under budget constraints (Crossa et al. 2017; Cobb et al. 2013; Yu et al. 2016; Fu et al. 2022). ML and DL models such as convolutional neural networks (CNNs), gradient boosting machines (GBMs), and random forests (RFs) have enhanced genomic prediction, outperforming traditional GS models including RR-BLUP and GBLUP in many crops for complex traits including yield, quality, and protein content (Sandhu et al. 2021; Montesinos-López et al. 2025; González-Camacho et al. 2018; Alemu et al. 2024). Easy-to-use online tools are now available to support GS using ML and DL models (Li et al. 2018b).

##### Deep learning empowers high-accuracy phenotyping

Deep learning (DL) has rapidly advanced phenotyping by enabling automated, high-throughput extraction of complex traits from images and sensor data. DL-based CNNs have achieved 96.7% accuracy in detecting Northern leaf blight in maize field images (DeChant et al. 2017). AI-driven platforms such as Leaf-GP and PlantCV extract trait data from aerial and ground imagery, accelerating breeding line evaluation (Araus et al. 2018; van Dijk et al. 2021). Integrated into tools such as EBS and BMS, DL facilitates large-scale, objective trait assessment and real-time decision-making in breeding pipelines (Eftekhari et al. 2024; MacNish et al. 2025).

##### Machine learning for predicting crop yields

ML models are increasingly used to predict crop yields by integrating AI-driven trait analysis (Van Klompenburg et al. 2020; Basir et al. 2021; MacNish et al. 2025). In rice, DL-based algorithms that combine image and feature data have accurately predicted yield (Zhou et al. 2025). However, in most public programs, the limited scale of trials and quantum of available data would remain major constraint for yield prediction using ML models.

##### AI-powered decision support systems

Rice breeding programs can now utilize open-source decision support systems and AI-powered platforms to improve strategies, optimize resources, and streamline trial design. By eliminating barriers of expertise, labor, and cost, these tools enable precise genotypic analysis, trait measurement, and yield prediction, steering in a new era of smart, efficient, and impact-driven breeding. Integrating AI, machine learning (ML), and deep learning (DL) across the pipeline transforms data into predictive power, accelerating decisions and enhancing precision and scalability under complex G × E interactions.

#### Strategic costing: optimizing investment in rice breeding

Strategic costing of rice breeding pipelines is fundamental to transitioning from conventional to modern, data-driven systems that enable smarter investments, optimized resource use, and greater pipeline efficiency and impact. By capturing full costs across key stages of the breeding pipeline (from crossing, rapid generation advancement to multi-environment testing, varietal release, and pre-commercial seed scaling activities), costing supports evidence-based investment planning, resource mobilization, and alignment with market demand and food security priorities. It helps identify cost bottlenecks, benchmark performance, and enhance operational efficiency through targeted interventions (Atlin et al. 2017a). Integrated with the breeder’s equation, costing allows simulation of genetic gain per unit cost, helping prioritize high-return scenarios, accelerate varietal turnover, and improve impact in climate-vulnerable regions (Gerald et al. 2013). Transparent, standardized costing also strengthens advocacy, supports public–private partnerships, and lays the foundation for sustainable breeding business models. Costing insights reveal core strengths, systemic inefficiencies, and key cost drivers shaping genetic gains, offering a data-driven basis for targeted investments to improve efficiency, sustainability, and impact.

Effective costing requires systematic pipeline mapping and disaggregation by activity (e.g., phenotyping, genotyping, and field management) and resource type (e.g., labor, consumables, and equipment). Activity-Based Costing (ABC) implemented with tools such as the University of Queensland’s Breeding Program Costing Tool (UQ-BPCT; https://aussorgm.org.au/downloads/breeding-costing-tool/) enables precise attribution of costs to functions and supports process optimization (Gourdji et al. 2015; Das et al. 2025). Scenario analysis enables comparison of pipeline architectures (e.g., SSD-RGA vs. pedigree, centralized vs. decentralized testing), aiding decisions on cost-effectiveness and scalability (Rutkoski 2022a; 2022b). We recently developed “a unified global costing framework for rice breeding (UGCF-Rice)”, a scalable model to assess and optimize the cost-efficiency of rice breeding pipelines globally, when used in combination with UQ-BPCT. We also demonstrated that strategic modernization can significantly improve efficiency, boost throughput, and minimize land use relative to conventional methods (Katiyar et al. unpublished). When linked with genetic gain and ROI projections, costing becomes a powerful tool to guide breeding modernization, accelerate impact, and ensure the long-term financial viability of rice breeding programs globally.

#### Resilient seed systems: driving faster varietal turnover

Transformation is only realized when resilient seed systems effectively translate breeding innovations into tangible impact. Genetic gains fulfill their purpose only when they reach farmers rapidly, equitably, and at scale, making seed systems the critical bridge between innovation and on-farm adoption, the final link in the transformation pathway. Accelerating the uptake of climate-resilient, market-preferred varieties requires inclusive, agile, and scalable delivery models driven by strong NARES leadership and dynamic value chain partnerships. A 50% increase in varietal turnover, with an average variety lifespan of less than 10 years, is vital for building resilient, competitive agri-food systems. This demands a paradigm shift toward pluralistic seed systems that fast-track varietal deployment through large-scale on-farm validation, farmer-preferred recommendations, visible promotion via crop cafeterias and head-to-head trials, rapid seed scale-up, and systematic withdrawal of outdated varieties. Stimulating demand is equally crucial through awareness campaigns, farmer-led demonstrations, and strong engagement with market stakeholders. Scalable innovations in seed health, multiplication, and last-mile delivery must ensure timely and affordable access to quality seed, particularly for smallholders. These efforts should be based in cost-effective, locally adapted models.

Real-time, low-cost ICT tools are necessary to monitor adoption, seed turnover, and information use on a large scale, enabling adaptive, data-driven responses. Most importantly, enabling policies are critical for streamlining variety release and deregistration, performance-based subsidies, and offering incentives for seed entrepreneurship and varietal replacement. Seed system transformation requires a dual drive-technical innovation coupled with institutional reform anchored in inclusive governance, strategic partnerships, and targeted capacity building (Text Box 3). The goal is faster, fairer, and broader access to improved varieties for smallholders, women, youth, and marginalized communities. Empowering NARES is critical, when local scientists and seed producers lead delivery, the right varieties reach farmers faster and have greater impact.

## TEXT Box 3: Revolving breeding into impact: five pillars of seed system reform

1. ***Strengthening early generation seed (EGS) supply:*** Guarantee timely, high-quality production of breeder and foundation seeds to eliminate downstream bottlenecks and support seamless varietal flow.
2. ***Inclusive, farmer-driven delivery models:*** Enhance adoption through participatory varietal selection, tricot on-farm trials, crop cafeterias, head-to-head trials and approaches responsive to gender, youth, market needs and agro-ecological diversity.
3. ***Decentralized multiplication and last-mile access:*** Expand local seed availability through community networks, Farmers Participatory Organizations, public–private partnership, and agri-entrepreneurs, ensuring affordable, timely access at scale.
4. ***Real-time monitoring and digital feedback:*** Use mobile, geo-referenced and ICT tools to track adoption, capture farmer feedback, and drive data-informed, adaptive scaling.
5. ***Enabling policies and institutional reform:*** Streamline variety release and retirement, incentivize varietal replacement, and align seed quality standards to accelerate delivery and impact.

### CRaFT-ABM: capacity and functional shifts for high-impact breeding

Rice breeding programs in developing countries face persistent challenges due to the slow and fragmented adoption of advanced technologies. Breeders operate with limited resources, restricted access to fit-for-purpose tools, inadequate infrastructure, and gaps in technical expertise. Critically, the capacity to integrate modern breeding principles, cutting-edge tools and technologies into efficient, data-driven breeding pipelines remains a major challenge for national programs. To address these systemic gaps, we introduce the “Capacity Reinforcement and Functional Transformation (CRaFT)” component under the Accelerated Breeding Modernization (ABM) framework, a strategic approach to simultaneously strengthen individual and institutional capacities, transforming rice breeding into a professional, high-performing system. Anchored in four pillars, including (i) human capital development, (ii) functional excellence through digital integration, (iii) institutional capacity building, and (iv) knowledge networks, CRaFT-ABM enables sustainable breeding modernization through inclusive, multi-directional capacity exchange. Grounded in co-development, mutual learning, and collaborative innovation, CRaFT-ABM positions NARES as equal partners in global rice breeding efforts toward achieving the SDGs by 2030.

Accelerated breeding modernization requires a shift from fragmented training efforts to a cohesive, inclusive model (Atlin et al 2017a; International Rice Research Institute 2022; CGIAR Excellence in Breeding 2022; CGIAR 2024a; 2024b; Katiyar et al. 2023a; 2023b). We propose the CRaFT-ABM platform (Capacity Reinforcement and Functional Transformation-Accelerated Breeding Modernization) as a strategic vehicle to integrate Breeding Excellence (BE) and Operational Excellence (OE) across NARES systems. CRaFT-ABM strengthens technical and non-technical capacities at all levels by embedding advanced breeding technologies, digital tools, robust data systems, and scalable delivery models. Additionally, CRaFT-ABM focuses on fostering leadership, soft skills, and continuous improvement. This holistic approach positions NARES not only to adopt innovation, but also to co-lead regional and global breeding agendas. Targeted investment in CRaFT-ABM will promote scalable innovation, enhance South–South and South–North collaboration, and build shared ownership of outcomes. A globally coordinated CRaFT-ABM platform will catalyze interconnected breeding networks, accelerating progress in agricultural transformation, food and nutrition security, and sustainable development. An overview of CRaFT, its goals and strategic interventions for ABM are provided in Table 5.

**Table 5:** ABM-CRaFT: empowering people, platforms, and institutions for breeding transformation

Capacity Goals	Key Interventions	Targeted impact
Technical excellence in accelerated breeding	Building breeder expertise in cutting-edge breeding pipelines, elite crosses, genomic tools, and speed breeding, via immersive, hands-on, and digital learning	• Data-driven breeding • Rapid BE/OE integration
Digital intelligence for breeding decisions	Strengthening capacity in smart breeding, digital platforms (BMS, EBS), FAIR-compliant data curation, and integrated electronic data capture and analytics (Field Book, BioFlow)	• Interoperable data, accurate by design • Streamlined trials, smarter decisions
Equipping breeders with cutting-edge predictive tools	• Optimizing Breeding Schemes via Simulation Tools • Genomic Selection with Predictive Breeding • Pipeline Costing and Strategic Investment Planning	• Streamlined pipeline architecture • Strategic resource optimization
Cultivate leadership and institutional agility	• Strengthening Leadership and Adaptive Capacity • Performance Diagnostics for Institutional Agility	• High-performing breeding teams • Agile and responsive institutions
Drive innovation through strategic alliances	• Joint Co-Innovation Platforms and Learning Alliances • Global/Regional Networks and South–South Collaboration	• Fast-track innovation adoption • Optimized, High-impact solutions
Institutionalize adaptive excellence	• Real-Time Feedback and Monitoring Systems • Participatory Planning and Iterative Evaluation Frameworks	• Adaptive, innovation-driven breeding programs • Sustained accelerated breeding modernization

## Concluding remarks and way forward

Accelerated Breeding Modernization-Breeding and Operational Excellence (ABM-BOx) is a transformative framework designed to revolutionize rice breeding systems into a high-performing, future-ready engine for food and climate security. It offers a globally scalable solution to enhance genetic gains, deliver climate-resilient, market-preferred varieties, and institutionalize impact at scale. For the first time, the rice breeding transformation agenda is strategically unified and operationalized, setting a global benchmark. ABM-BOx integrates two synergistic pillars: A. Breeding Excellence (BE) enhances selection accuracy, intensity, and genetic variance through demand-driven breeding, strategic parental selection, recurrent population breeding, breeding scheme optimization, and genomic selection. B. Operational Excellence (OE) compresses cycle time, improves accuracy and efficiency through speed breeding, smart breeding-digital tools, breeding informatics-AI-powered decision tools, strategic costing, and resilient seed system. Together, BE and OE re-engineer breeding pipelines for speed, efficiency, and scalability (Fig. 3).

**Fig. 6:**
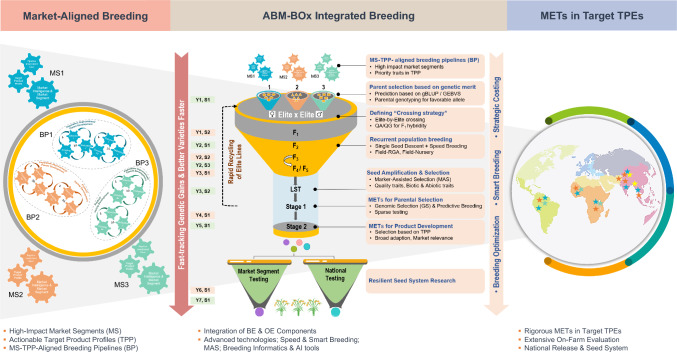
ABM***-BOx***: End-to-End Modernization of Rice Breeding Systems. This figure illustrates the integration of the *ABM-BOx* framework to transform rice breeding for scale, speed, and impact. Left: Pipelines are aligned with market insight by linking priority market segments (MS) to actionable target product profiles (TPPs). Center: Accelerated Breeding Modernization-Breeding and Operational Excellence (*ABM-BOx*) components, including global science, strategies, cutting-edge tools, innovations, and global best practices are embedded to modernize breeding workflows. Right: Multi-environment trials (METs) across target environments (TPEs) ensure selection for stability, adaptation, and relevance. Anchored in SDGs 1, 2, and 13, *ABM-BOx* enables climate-smart, high-impact rice breeding

**Fig. 7:**
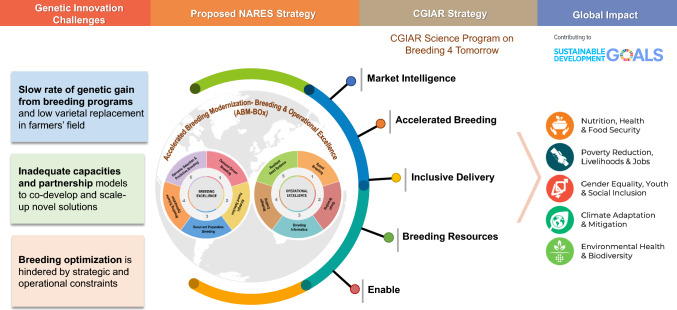
NARES-CGIAR Breeding Convergence for Global Impact. The figure highlights major challenges in CGIAR breeding: slow genetic gains, limited capacity, and systemic inefficiencies and the strategic response through the Breeding 4 Tomorrow global science program. We present how integration of the *ABM-BOx* framework can transform national programs, align them with global breeding efforts, and accelerate impact across key SDGs, including food security, climate resilience, poverty reduction, gender equity, and biodiversity

To catalyze a step-change in genetic gains and climate resilience, rice breeding programs must urgently embrace a bold, actionable, and future-ready modernization agenda. First, institutionalize the ABM-BOx engine across rice breeding programs by embedding the integrated Breeding-Operational Excellence (BE-OE) engine at the heart of breeding pipelines to accelerate precision, efficiency, and impact (Fig. 6). Second, fully transition to demand-driven, TPP-anchored breeding by identifying high-impact market segments, designing actionable TPPs, and aligning breeding pipelines with market insights. Third, future-proof breeding through recurrent population improvement and genomic selection, leveraging shortened breeding cycles, improved selection intensity, and building pipelines adaptive to shifting trait targets. Fourth, drive efficiency by optimizing breeding schemes using simulation-driven approaches to maximize genetic gain per dollar, right-size trials, and enable outcome-based investment planning. Fifth, mainstream speed, smart, and AI-powered breeding by integrating rapid generation advance to compress cycle time. Utilize smart breeding and AI-driven prediction tools to scale precision, improve prediction accuracy, and enhance decision-making across environments. Finally, treat seed systems as co-creators of impact by embedding ICT-enabled, decentralized, and resilient seed systems into breeding pipelines. This will accelerate varietal turnover, ensure quality assurance, and deliver improved varieties swiftly to farmers through robust delivery networks. Aligned with SDGs 1, 2, and 13, ABM-BOx offers a mission-critical strategy to modernize breeding and build resilient, inclusive food systems. However, implementation of the ABM-BOx recommendations by NARES may be constrained by funding gaps, rigid governance, limited technical capacity, and inadequate digital infrastructure. Unlocking its full potential of ABM-BOx demands a mindset change, sustained investment, supportive policy frameworks, and coordination across national and global networks to deliver high-impact, climate-smart breeding at scale (Fig. 7).

## References

[CR1] Adjah KL, Asante MD, Toure A, Aziadekey M, Amoako-Andoh FO, Frei M, Diallo Y, Agboka K (2022) Improvement of rice production under drought conditions in West Africa: application of QTLs in breeding for drought resistance. Rice Sci 29:512–521

[CR2] Ahmed MME, Biswas PS, Afrin W, Khan MY, Sarker MRA, Iftekharuddaula KM (2022) Recent advances in population improvement through RGA under irrigated Boro rice breeding program in Bangladesh. BRJ 26:33–46

[CR3] Alemu A, Åstrand J, Montesinos-Lopez OA, Sanchez JI Y, Fernandez-Gonzalez J, Tadesse W, Vetukuri RR, Carlsson AS, Ceplitis A, Crossa J, Ortiz R, Chawade A (2024) Genomic selection in plant breeding: Key factors shaping two decades of progress. Mol Plant 17:552–578 38475993 10.1016/j.molp.2024.03.007

[CR4] Allard RW (1999) History of plant population genetics. Annu Rev Genet 33:1–27 10690402 10.1146/annurev.genet.33.1.1

[CR5] Araus JL, Kefauver SC, Zaman-Allah M, Olsen MS, Cairns JE (2018) Translating high-throughput phenotyping into genetic gain. Trends Plant Sci 23:451–466 29555431 10.1016/j.tplants.2018.02.001 PMC5931794

[CR6] Arbelaez JD, Dwiyanti MS, Tandayu E, Llantada K, Jarana A, Ignacio JC, Platten JD, Cobb J, Rutkoski JE, Thomson MJ, Kretzschmar T (2019) 1k-RiCA (1K-Rice Custom Amplicon) a novel genotyping amplicon-based SNP assay for genetics and breeding applications in rice. Rice 12:55 31350673 10.1186/s12284-019-0311-0 PMC6660535

[CR7] Atlin GN, Econopouly BF (2022) Simple deterministic modeling can guide the design of breeding pipelines for self-pollinated crops. Crop Sci 62:661–678

[CR8] Atlin GN, Cairns JE, Das B (2017a) Rapid breeding and varietal replacement are critical to adaptation of cropping systems in the developing world to climate change. Glob Food Secur 12:31–37 10.1016/j.gfs.2017.01.008 PMC5439485 28580238

[CR9] Atlin GN, Cairns JE, Das B (2017b) Rapid breeding and the molecular revolution. Nat Plants 3:1708128628132

[CR10] Bartholome J, Prakash PT, Cobb JN (2022) Genomic prediction: progress and perspectives for rice improvement. In Genomic Predict complex Traits: Methods Protoc. 10.1007/978-1-0716-2205-6_21 10.1007/978-1-0716-2205-6_21 35451791

[CR11] Basir MS, Chowdhury M, Islam MN, Ashik-E-Rabbani M (2021) Artificial neural network model in predicting yield of mechanically transplanted rice from transplanting parameters in Bangladesh. J Agric Food Res 5:100186

[CR116] Bassi FM, Bentley AR, Charmet G, Ortiz R, Crossa J (2016) Breeding schemes for the implementation of genomic selection in wheat (*Triticum* spp.). Plant Sci 242:23–36 26566822 10.1016/j.plantsci.2015.08.021

[CR12] Bernardo R (2020) Reinventing quantitative genetics for plant breeding: something old, something new, something borrowed, something BLUE. Heredity 125:375–385 32296132 10.1038/s41437-020-0312-1 PMC7784685

[CR13] Bhat JA, Feng X, Mir ZA, Raina A, Siddique KH (2023) Recent advances in artificial intelligence, mechanistic models, and speed breeding offer exciting opportunities for precise and accelerated genomics-assisted breeding. Physiol Plant 175:e13969 37401892 10.1111/ppl.13969

[CR14] Bhosale S, Thathapalli Prakash P, Kadaru SB, Hussain W, Dixit S, Ali J, Platten JD, Islam MR, Singh VK, Murori R, Ndayiragije A, Panchbhai A, Nayak S, Dela Cruz P, Demont M, Bartholome J, Covarrubias-Pazaran G, Cobb JN, Bhardwaj H (2025) Onerice breeding framework: an end-to-end system to develop better varieties faster. Crop Sci 65:e70087. 10.1002/csc2.70087

[CR15] Bin Rahman AR, Zhang J (2023) Trends in rice research: 2030 and beyond. Food Energy Secur 12:e390

[CR16] Boyer JS, Byrne P, Cassman KG, Cooper M, Delmer D, Greene T, Gruis F, Habben J, Hausmann N, Kenny N, Lafitte R, Paszkiewicz S, Porter D, Schlegel A, Schussler J, Setter T, Shanahan J, Sharp RE, Vyn TJ, Warner D, Gaffney J (2013) The US drought of 2012 in perspective: a call to action. Glob Food Secur 2:139–143

[CR17] CGIAR Excellence in Breeding (2022) Breeding program modernization: Empowering NARES to transform breeding in East and Southern Africa. https://excellenceinbreeding.org/news/breeding-program-modernization-empowering-nares-transform-breeding-east-and-southern-africa Accessed 19 Jun 2025

[CR18] CGIAR Excellence in Breeding (2023, August 14) Fostering food security and climate resilience: Accelerated breeding modernization to support hi-impact NARES in Nepal. https://excellenceinbreeding.org/news/fostering-food-security-and-climate-resilience-accelerated-breeding-modernization-support-hi Accessed 19 Jun 2025

[CR19] CGIAR Initiative on Accelerated Breeding. (2023, July 5) Empowering NARES for high-impact rice breeding in Sub-Saharan Africa. https://www.cgiar.org/news-events/news/empowering-nares-for-high-impact-rice-breeding-in-sub-saharan-africa/ Accessed 19 Jun 2025

[CR20] CGIAR Initiative on Accelerated Breeding (2025) CGIAR Research Initiative on Accelerated Breeding: Annual Technical Report 2024. Montpellier, France: CGIAR System Organization. https://hdl.handle.net/10568/174241 Accessed 12 Jul 2025

[CR21] CGIAR (2023) Global Market Intelligence Platform (GloMIP). https://www.cgiar.org/initiative-result/global-market-intelligence-platform-glomip/

[CR22] CGIAR (2024a) Breeding for Tomorrow Program: Full design document. Agenda item SC21–05a, 21st CGIAR System Council meeting, Berlin, Germany, 11–12 December 2024. Montpellier: CGIAR. https://hdl.handle.net/10568/162806

[CR23] CGIAR (2024b) Capacity Sharing Accelerator: Full design document. Agenda item SC21–05a, 21st CGIAR System Council meeting, Berlin, Germany, 11–12 December 2024. Montpellier: CGIAR. https://hdl.handle.net/10568/162807

[CR24] Cheng Q, Wang X (2024) Machine learning for AI breeding in plants. Genomics Proteomics Bioinf 22:qzae051 10.1093/gpbjnl/qzae051 PMC11479635 38954837

[CR25] Chung PY, Liao CT (2020) Identification of superior parental lines for biparental crossing via genomic prediction. PLoS ONE 15:e0243159 33270706 10.1371/journal.pone.0243159 PMC7714229

[CR26] Clark SA, van der Werf J (2013) Genomic best linear unbiased prediction (gBLUP) for the estimation of genomic breeding values. Genome-wide association studies and genomic prediction. Humana Press, Totowa NJ, pp 321–330 10.1007/978-1-62703-447-0_13 23756897

[CR27] Cobb JN, DeClerck G, Greenberg A, Clark R, McCouch S (2013) Next-generation phenotyping: requirements and strategies for enhancing our understanding of genotype–phenotype relationships and its relevance to crop improvement. Theor Appl Genet 126:867–887 23471459 10.1007/s00122-013-2066-0 PMC3607725

[CR28] Cobb JN, Juma RU, Biswas PS, Arbelaez JD, Rutkoski J, Atlin G, Hagen T, Quinn M, Ng EH (2019) Enhancing the rate of genetic gain in public-sector plant breeding programs: lessons from the breeder’s equation. Theor Appl Genet 132:627–645 30824972 10.1007/s00122-019-03317-0 PMC6439161

[CR29] Collard BC, Beredo JC, Lenaerts B, Mendoza R, Santelices R, Lopena V, Verdeprado H, Raghavan C, Gregorio GB, Vial L, Demont M, Partha SB, Iftekharuddaula KM, Rahman MA, Cobb JN, Islam MR (2017) Revisiting rice breeding methods–evaluating the use of rapid generation advance (RGA) for routine rice breeding. Plant Prod Sci 20:337–352

[CR30] Collard BC, Gregorio GB, Thomson MJ, Islam R, Vergara GV, Laborte AG, Nissila E, Kretzschmar T, Cobb JN (2019) Transforming rice breeding redesigning the irrigated breeding pipeline at the International Rice Research Institute (IRRI). Crop Breed Genet Genomics. 10.20900/cbgg20190008

[CR31] Covarrubias-Pazaran G, Martini JW, Quinn M, Atlin G (2021) Strengthening public breeding pipelines by emphasizing quantitative genetics principles and open-source data management. Front Plant Sci 12:681624 34326855 10.3389/fpls.2021.681624 PMC8313805

[CR32] Covarrubias-Pazaran G, Gebeyehu Z, Gemenet D, Werner C, Labroo M, Sirak S, Coaldrake P, Rabbi I, Kayondo SI, Parkes E, Kanju E, Mbanjo EGN, Agbona A, Kulakow P, Quinn M, Debaene J (2022) Breeding schemes: what are they, how to formalize them, and how to improve them? Front Plant Sci 12:791859 35126417 10.3389/fpls.2021.791859 PMC8813775

[CR33] Crossa J, Pérez-Rodríguez P, Cuevas J, Montesinos-López O, Jarquín D, De Los Campos G, Burgueño J, González-Camacho JM, Pérez-Elizalde S, Beyene Y, Dreisigacker S, Singh R, Zhang X, Gowda M, Roorkiwal M, Rutkoski J, Varshney RK (2017) Genomic selection in plant breeding: methods, models, and perspectives. Trends Plant Sci 22:961–975 28965742 10.1016/j.tplants.2017.08.011

[CR34] Crossa J, Martini JW, Vitale P, Pérez-Rodríguez P, Costa-Neto G, Fritsche-Neto R, Runcie D, Cuevas J, Toledo F, Li H, De Vita P, Gerard G, Dreisigacker S, Crespo-Herrera L, Pierre CS, Bentley A, Lillemo M, Ortiz R, Montesinos-López OA, Montesinos-López, A (2025) Expanding genomic prediction in plant breeding: harnessing big data, machine learning, and advanced software. Trends Plant Sci 30:756–774 39890501 10.1016/j.tplants.2024.12.009

[CR35] Custodio MC, Demont M, De Steur H (2023) Market intelligence for guiding crop improvement: a systematic review of stakeholder preference studies in the rice sector in the Global South and beyond. Compr Rev Food Sci Food Saf 22:4404–4432 37602888 10.1111/1541-4337.13228

[CR36] Dai S, Shulski MD, Hubbard KG, Takle ES (2015) A spatiotemporal analysis of Midwest US temperature and precipitation trends during the growing season from 1980 to 2013. Int J Climatol 36:517–525

[CR37] Das B, Mutiga SK, Odiyo O, Madahana S, Milic D, Sinyinda L, Mwansa K, Mukaro R, Chaingeni D, Asea G, Kwemoi DB, Musundire L (2025) Costing of the breeding operations for the national maize programs in Eastern and Southern Africa. Front Sustain Food Syst 9:1545600

[CR38] DeChant C, Wiesner-Hanks T, Chen S, Stewart EL, Yosinski J, Gore MA, Nelson RJ, Lipson H (2017) Automated identification of northern leaf blight-infected maize plants from field imagery using deep learning. Phytopathology 107:1426–1432 28653579 10.1094/PHYTO-11-16-0417-R

[CR39] Desta ZA, Ortiz R (2014) Genomic selection: genome-wide prediction in plant improvement. Trends Plant Sci 19:592–601 24970707 10.1016/j.tplants.2014.05.006

[CR40] Donovan J, Coaldrake P, Rutsaert P, Bänzinger M, Gitonga A, Naziri D, Demont M, Newby J, Ndegwa M (2022) Market intelligence for informing crop-breeding decisions by CGIAR and NARES. Market Intelligence Brief Series 1. CGIAR, Montpellier

[CR41] Eftekhari M, Ma C, Orlov YL (2024) Applications of artificial intelligence, machine learning, and deep learning in plant breeding. Front Plant Sci 15:1420938 38841285 10.3389/fpls.2024.1420938 PMC11150839

[CR42] Endelman JB, Atlin GN, Beyene Y, Semagn K, Zhang X, Sorrells ME, Jannink JL (2014) Optimal design of preliminary yield trials with genome-wide markers. Crop Sci 54:48–59

[CR43] Falconer DS (1996) Introduction to quantitative genetics. Pearson Education India

[CR44] Fang H, Zhang Q, Zhang S, Zhang T, Pan F, Cui Y, Thomsen ST, Jakobsen LS, Liu A, Pires SM (2021) Risk–benefit assessment of consumption of rice for adult men in China. Front Nutr 8:694370 34368209 10.3389/fnut.2021.694370 PMC8342936

[CR45] FAO (2017) The Future of food and agriculture – trends and challenges. http://www.fao.org/3/i6583e/i6583e.pdf

[CR46] FAO, IFAD, UNICEF, WFP and WHO (2024) The state of food security and nutrition in the world 2024 – Financing to end hunger, food insecurity and malnutrition in all its forms. Rome. 10.4060/cd1254en

[CR47] USDA FAS (2024) Grain: world markets and trade. Foreign agricultural service

[CR48] Food and agriculture organization of the united nations (2022) rice -a sector of opportunity. In South–South and triangular cooperation in Sub-Saharan Africa: Case studies (Chapter 3). https://openknowledge.fao.org/server/api/core/bitstreams/e550008c-f9ba-4a12-adf3-656355765bf5/content/ssc-sub-saharan-africa-2022/chapter-3.html

[CR49] Food and agriculture organization of the united nations (2025) FAOSTAT statistical database. Rome. Accessed 12 Jul 2025

[CR50] Friedmann M, Chaudari S, Mendes T, Polar V, Ssali RT (2024). Evolution of market segmentation and target product profile development in the CIP potato and sweet potato breeding programs. 10.4160/cip.2024.10.002

[CR51] Fu J, Hao Y, Li H, Reif JC, Chen S, Huang C, Wang G, Li X, Xu Y, Li L (2022) Integration of genomic selection with doubled-haploid evaluation in hybrid breeding: from GS 1.0 to GS 4.0 and beyond. Mol Plant 15:577–580 35149251 10.1016/j.molp.2022.02.005

[CR52] Gaynor RC, Gorjanc G, Bentley AR, Ober ES, Howell P, Jackson R, Mackay IJ, Hickey JM (2017) A two-part strategy for using genomic selection to develop inbred lines. Crop Sci 57:2372–2386

[CR53] Gaynor RC, Gorjanc G, Hickey JM (2021) AlphaSimR: an R package for breeding program simulations. G3 Genes|genomes|genetics 11:jkaa017 33704430 10.1093/g3journal/jkaa017 PMC8022926

[CR54] Gerald NDLF, Frei UK, Lübberstedt T (2013) Accelerating plant breeding. Trends Plant Sci 18:667–672 24080381 10.1016/j.tplants.2013.09.001

[CR55] Gilmour AR, Cullis BR, Verbyla AP, Verbyla AP (1997) Accounting for natural and extraneous variation in the analysis of field experiments. J Agric Biol Environ Stat 2:269. 10.2307/1400446

[CR56] González-Camacho JM, Ornella L, Pérez-Rodríguez P, Gianola D, Dreisigacker S, Crossa J (2018) Applications of machine learning methods to genomic selection in breeding wheat for rust resistance. Plant Genome 11:170104 10.3835/plantgenome2017.11.0104 PMC12962436 30025028

[CR57] Gorjanc G, Gaynor RC, Hickey JM (2018) Optimal cross selection for long-term genetic gain in two-part programs with rapid recurrent genomic selection. Theor Appl Genet 131:1953–1966 29876589 10.1007/s00122-018-3125-3 PMC6096640

[CR58] Gourdji SM, Sibley AM, Lobell DB (2015) Advances in agriculture and climate change adaptation. Environ Res Lett 10:123003

[CR59] Henderson CR (1975) Best linear unbiased estimation and prediction under a selection model. Biometrics. 10.2307/2529430 1174616

[CR117] Henryon M, Berg P, Sørensen AC (2014) Animal-breeding schemes using genomic information need breeding plans designed to maximise long-term genetic gains. Livest Sci 166:38–47

[CR60] Hossain F, Yadava DK, Singh AK (2025) Role of public-funded research in development of seed sector in India. Indian seed sector: evolution, technology trade and impact. Springer Nature Singapore, Singapore, pp 91–113

[CR61] International Rice Research Institute (2022, December 13) NARES-CGIAR global networks adapt breeding program modernization in Asia and Sub-Saharan Africa. https://www.irri.org/news-and-events/news/nares-cgiar-global-networks-adapt-breeding-program-modernization-asia-and-sub Accessed 19 Jun 2025

[CR62] Jangra S, Chaudhary V, Yadav RC, Yadav NR (2021) High-throughput phenotyping: a platform to accelerate crop improvement. Phenomics 1:31–53 36939738 10.1007/s43657-020-00007-6 PMC9590473

[CR63] Jarquin D, Howard R, Crossa J, Beyene Y, Gowda M, Martini JW, Covarrubias Pazaran G, Burgueño J, Pacheco A, Grondona M, Wimmer V (2020) Genomic prediction enhanced sparse testing for multi-environment trials. G3 Genes|genomes|genetics 10:2725–2739 32527748 10.1534/g3.120.401349 PMC7407457

[CR64] Juma RU, Bartholomé J, Thathapalli Prakash P, Hussain W, Platten JD, Lopena V, Verdeprado H, Murori R, Ndayiragije A, Katiyar SK, Islam MR, Biswas PS, Rutkoski JE, Arbelaez JD, Mbute FN, Miano DW, Cobb JN (2021) Identification of an elite core panel as a key breeding resource to accelerate the rate of genetic improvement for irrigated rice. Rice 14:92 34773509 10.1186/s12284-021-00533-5 PMC8590642

[CR65] Katiyar S K, Das R R, Bilaro A, Asante M, Abade H, Diop B, Chizhande N, Yeboah A, Murori R, Ndayiragije A, Raharinivo V, Iftekharuddaula K, Islam M, Yadaw R, Sood S, Singh A, Chandel G, Verma R, Srinivas T, Satish Y, Chandramohan Y, Ng EH, Bhosale S, Kadaru S, Musundire L, Das B (2023b, October 23) Accelerated breeding modernization: Transforming NARES for driving Sub-Saharan Africa’s food security [Plenary talk]. 3rd African Plant Breeders Association (APBA) Conference, Mohammed VI Polytechnic University, Benguerir, Morocco. https://www.cgiar.org/news-events/event/3rd-african-plant-breeders-association-apba-conference/ Accessed 19 Jun 2025

[CR66] Katiyar S K, Das R R, Bilaro A, Asante M, Abade H, Diop B, Chizhande N, Yeboah A, Murori R, Ndayiragije A, Raharinivo V, Iftekharuddaula K, Islam M, Yadaw R, Sood S, Singh A, Chandel G, Verma R, Srinivas T, Satish Y, Chandramohan Y, Ng EH, Kadaru S, Bhosale S, Musundire L, Das B (2023a) Revitalizing NARES: Accelerated Breeding Modernization for Enhanced Genetic Gain and Ensured Food Security Amid Climate Flux. In 6th International Rice Congress 2023: Accelerating Transformation of Rice-Based Food Systems: From Gene to Globe (Abstract No. 324). https://di9mr54a05a64.cloudfront.net/api-dlg.expoplatform.com/files/Abstracts/abstract-324.pdf Accessed 19 Jun 2025

[CR115] Katiyar SK, Das RR, Ramayya PJ (2025) Accelerating rice breeding modernization through CGIAR–NARES partnerships. Figshare. 10.6084/m9.figshare.30363571

[CR67] Kruseman G, Mottaleb KA, Tesfaye K, Bairagi S, Robertson R, Mandiaye D, Frija A, Gbegbelegbe S, Alene A, Prager S (2020) Rural transformation and the future of cereal-based agri-food systems. Glob Food Secur 26:100441

[CR68] Kusmec A, Zheng Z, Archontoulis S, Ganapathysubramanian B, Hu G, Wang L, Yu J, Schnable PS (2021) Interdisciplinary strategies to enable data-driven plant breeding in a changing climate. One Earth 4:372–383

[CR69] Lehermeier C, Teyssèdre S, Schön CC (2017) Genetic gain increases by applying the usefulness criterion with improved variance prediction in selection of crosses. Genetics 207:1651–1661 29038144 10.1534/genetics.117.300403 PMC5714471

[CR70] Lenaerts S, Van Der Borght M, Callens A, Van Campenhout L (2018) Suitability of microwave drying for mealworms (*Tenebrio molitor*) as alternative to freeze drying: impact on nutritional quality and colour. Food Chem 254:129–136 29548432 10.1016/j.foodchem.2018.02.006

[CR71] Li H, Rasheed A, Hickey LT, He Z (2018) Fast-forwarding genetic gain. Trends Plant Sci 23(3):184–186 29426713 10.1016/j.tplants.2018.01.007

[CR72] Li B, Zhang N, Wang YG, George AW, Reverter A, Li Y (2018b) Genomic prediction of breeding values using a subset of SNPs identified by three machine learning methods. Front Genet 9:237 30023001 10.3389/fgene.2018.00237 PMC6039760

[CR73] Li J, Zhang D, Yang F, Zhang Q, Pan S, Zhao X, Zhang Q, Han Y, Yang J, Wang K, Zhao C (2024) TrG2P: a transfer-learning-based tool integrating multi-trait data for accurate prediction of crop yield. Plant Commun. 10.1016/j.xplc.2024.100975 38751121 10.1016/j.xplc.2024.100975 PMC11287160

[CR74] Lingwal S, Bhatia KK, Singh M (2022) A novel machine learning approach for rice yield estimation. J Exp Theor Artif Intell 36:337–356. 10.1080/0952813X.2022.2062458

[CR75] Liu Y, Wang D, He F, Wang J, Joshi T, Xu D (2019) Phenotype prediction and genome-wide association study using deep convolutional neural network of soybean. Front Genet 10:1091 31824557 10.3389/fgene.2019.01091 PMC6883005

[CR76] Lobos GA, Camargo AV, Del Pozo A, Araus JL, Ortiz R, Doonan JH (2017) Plant phenotyping and phenomics for plant breeding. Front Plant Sci 8:2181 29375593 10.3389/fpls.2017.02181 PMC5770690

[CR77] Ma W, Qiu Z, Song J, Li J, Cheng Q, Zhai J, Ma C (2018) A deep convolutional neural network approach for predicting phenotypes from genotypes. Planta 248:1307–1318 30101399 10.1007/s00425-018-2976-9

[CR78] Ma X, Wang H, Wu S, Han B, Cui D, Liu J, Zhang Q, Xia X, Song P, Tang C, Geng L (2024) Deepccr: Large-scale genomics-based deep learning method for improving rice breeding. Plant Biotechnol J 22:2691 38805625 10.1111/pbi.14384 PMC11536438

[CR79] MacNish TR, Danilevicz MF, Bayer PE, Bestry MS, Edwards D (2025) Application of machine learning and genomics for orphan crop improvement. Nat Commun 16:982 39856113 10.1038/s41467-025-56330-x PMC11760368

[CR80] Merugumala GR, Pv S, Narne C, Bnvsr R, Pv RR, V D (2019) Molecular breeding of “Swarna”, a mega rice variety for lodging resistance. Mol Breed 39:55

[CR81] Meuwissen TH, Hayes BJ, Goddard M (2001) Prediction of total genetic value using genome-wide dense marker maps. Genetics 157:1819–1829 11290733 10.1093/genetics/157.4.1819 PMC1461589

[CR82] Mokhtar A, He H, Nabil M, Kouadri S, Salem A, Elbeltagi A (2024) Securing China’s rice harvest: unveiling dominant factors in production using multi-source data and hybrid machine learning models. Sci Rep 14:14699 38926368 10.1038/s41598-024-64269-0 PMC11208568

[CR83] Montesinos-López A, Montesinos-López OA, Ramos-Pulido S, Mosqueda-González BA, Guerrero-Arroyo EA, Crossa J, Ortiz R (2025) Artificial intelligence meets genomic selection: comparing deep learning and GBLUP across diverse plant datasets. Front Genet 16:1568705 40364946 10.3389/fgene.2025.1568705 PMC12069277

[CR84] Mrode R (2014) Genetic covariance between relatives. Linear models for the prediction of animal breeding values. CABI, Wallingford UK, pp 22–33

[CR85] Nguyen VH, Morantte RIZ, Lopena V, Verdeprado H, Murori R, Ndayiragije A, Katiyar SK, Islam MR, Juma RU, Flandez-Galvez H, Glaszmann JC, Cobb JN, Bartholomé J (2023) Multi-environment genomic selection in rice elite breeding lines. Rice 16:7 36752880 10.1186/s12284-023-00623-6 PMC9908796

[CR86] Piepho HP, Möhring J, Melchinger AE, Büchse A (2008) Blup for phenotypic selection in plant breeding and variety testing. Euphytica 161:209–228

[CR87] Pook T, Tost ML, Simianer H (2025) Optimization of recurrent rapid cycle breeding in maize for sustained long-term genetic improvement via stochastic simulations. G3: Genes, Genomes, Genetics. 10.1093/g3journal/jkaf100 40392147 10.1093/g3journal/jkaf100 PMC12239619

[CR88] Ray DK, Mueller ND, West PC, Foley JA (2013) Yield trends are insufficient to double global crop production by 2050. PLoS ONE 8:e66428 23840465 10.1371/journal.pone.0066428 PMC3686737

[CR89] Rebetzke G, Jimenez-Berni J, Fischer R, Deery D, Smith D (2018) High-throughput phenotyping to enhance the use of crop genetic resources. Plant Sci 282:40–48. 10.1016/j.plantsci.2018.06.017 31003610 10.1016/j.plantsci.2018.06.017

[CR90] Rife TW, Poland JA (2014) Field book: an open-source application for field data collection on android. Crop Sci 54:1624–1627

[CR91] Rutkoski JE (2019) a practical guide to genetic gain. Adv Agron 157:217–249. 10.1016/bs.agron.2019.05.001

[CR92] Rutkoski JE (2019b) Estimation of realized rates of genetic gain and indicators for breeding program assessment. Crop Sci 59:981–993

[CR93] Rutkoski JE, Krause MR, Sorrells ME (2022a) Breeding methods: Line development. Wheat improvement: Food security in a changing climate. Springer International Publishing, Cham, pp 69–82

[CR94] Rutkoski JE, Krause MR, Sorrells ME (2022b) Breeding methods: population improvement and selection methods. wheat improvement: food security in a changing climate. Springer International Publishing, Cham, pp 83–96

[CR95] Samal P, Babu SC, Mondal B, Mishra SN (2022) The global rice agriculture towards 2050: an inter-continental perspective. Outlook Agric 51:164–172. 10.1177/00307270221088338

[CR96] Sandhu KS, Lozada DN, Zhang Z, Pumphrey MO, Carter AH (2021) Deep learning for predicting complex traits in spring wheat breeding program. Front Plant Sci 11:613325 33469463 10.3389/fpls.2020.613325 PMC7813801

[CR97] Sarkar TK, Roy DK, Kang YS, Jun SR, Park JW, Ryu CS (2024) Ensemble of machine learning algorithms for rice grain yield prediction using UAV-based remote sensing. J Biosyst Eng 49:1–19

[CR98] Seck PA, Diagne A, Mohanty S, Wopereis MC (2012) Crops that feed the world 7: rice. Food Secur 4:7–24

[CR99] Seck F, Covarrubias-Pazaran G, Gueye T, Bartholomé J (2023) Realized genetic gain in rice: achievements from breeding programs. Rice 16:61 38099942 10.1186/s12284-023-00677-6 PMC10724102

[CR100] Seck F, Prakash PT, Covarrubias-Pazaran G, Gueye T, Diédhiou I, Bhosale S, Kadaru S, Bartholomé J (2024) Stochastic simulation to optimize rice breeding at IRRI. Front Plant Sci 15:1488814 39554523 10.3389/fpls.2024.1488814 PMC11563958

[CR101] Sonnino A, Dhlamini Z, Mayer-Tasch L, Santucci FM (2009) Assessing the socio-economic impacts of non-transgenic biotechnologies in developing countries. Socio-economic impacts of non-transgenic biotechnologies in developing countries: The case of plant micropropagation in Africa. FAO. ftp://ftp. fao. org/docrep/fao/011/i0340e/i0340e. pdf.

[CR102] Tanaka J, Hayashi T, Iwata H (2016) A practical, rapid generation-advancement system for rice breeding using simplified biotron breeding system. Breed Sci 66:542–551. 10.1270/jsbbs.15038 27795679 10.1270/jsbbs.15038 PMC5010295

[CR103] United Nations (2023) SDG Summit 2023. https://www.un.org/en/conferences/SDGSummit2023

[CR104] van Dijk Aalt Dirk Jan, Gert Kootstra, Willem Kruijer, Dick de Ridder (2021) Machine learning in plant science and plant breeding. Iscience 24(1) 10.1016/j.isci.2020.101890 PMC7750553 33364579

[CR105] Van Klompenburg T, Kassahun A, Catal C (2020) Crop yield prediction using machine learning: a systematic literature review. Comput Electron Agric 177:105709

[CR106] Voss-Fels KP, Cooper M, Hayes BJ (2019) Accelerating crop genetic gains with genomic selection. Theor Appl Genet 132:669–686 30569365 10.1007/s00122-018-3270-8

[CR107] Wu H, Han R, Zhao L, Liu M, Chen H, Li W, Li L (2025) AutoGP: an intelligent breeding platform for enhancing maize genomic selection. Plant Commun. 10.1016/j.xplc.2025.101240 39789848 10.1016/j.xplc.2025.101240 PMC12010379

[CR108] Xu Y, Zhang X, Li H, Zheng H, Zhang J, Olsen MS, Varshney RK, Prasanna BM, Qian Q (2022) Smart breeding driven by big data, artificial intelligence, and integrated genomic-enviromic prediction. Mol Plant 15:1664–1695 36081348 10.1016/j.molp.2022.09.001

[CR109] Yabe S, Iwata H, Jannink JL (2017) A simple package to script and simulate breeding schemes: the breeding scheme language. Crop Sci 57:1347–1354

[CR110] Yang W, Feng H, Zhang X, Zhang J, Doonan JH, Batchelor WD, Xiong L, Yan J (2020) Crop phenomics and high-throughput phenotyping: past decades, current challenges, and future perspectives. Mol Plant 13:187–214 31981735 10.1016/j.molp.2020.01.008

[CR111] Yang W, Guo T, Luo J, Zhang R, Zhao J, Warburton ML, Xiao Y, Yan J (2022) Target-oriented prioritization: targeted selection strategy by integrating organismal and molecular traits through predictive analytics in breeding. Genome Biol 23:80 35292095 10.1186/s13059-022-02650-w PMC8922918

[CR112] Yoosefzadeh Najafabadi M, Hesami M, Eskandari M (2023) Machine learning-assisted approaches in modernized plant breeding programs. Genes 14:777 37107535 10.3390/genes14040777 PMC10137951

[CR113] Yu X, Li X, Guo T, Zhu C, Wu Y, Mitchell SE, Roozeboom KL, Wang D, Wang ML, Pederson GA, Tesso TT (2016) Genomic prediction contributing to a promising global strategy to turbocharge gene banks. Nat Plants 2:1–7 10.1038/nplants.2016.150 27694945

[CR114] Zhou H, Huang F, Lou W, Gu Q, Ye Z, Hu H, Zhang X (2025) Yield prediction through UAV-based multispectral imaging and deep learning in rice breeding trials. Agric Syst 223:104214

